# Associations of Dietary Patterns and Metabolic-Hormone Profiles with Breast Cancer Risk: A Case-Control Study

**DOI:** 10.3390/nu10122013

**Published:** 2018-12-19

**Authors:** Beata Krusinska, Lidia Wadolowska, Malgorzata Anna Slowinska, Maciej Biernacki, Marek Drozdowski, Tomasz Chadzynski

**Affiliations:** 1 Department of Human Nutrition, University of Warmia and Mazury in Olsztyn, Sloneczna 45f, 10-718 Olsztyn, Poland; lidia.wadolowska@uwm.edu.pl (L.W.); malgorzata.slowinska@uwm.edu.pl (M.A.S.); tomasz.chadzynski@uwm.edu.pl (T.C.); 2 Department of Surgery, University of Warmia and Mazury in Olsztyn, 11-041 Olsztyn, Poland; maciej.biernacki@uwm.edu.pl; 3 Department of Laboratory Medicine, University of Warmia and Mazury in Olsztyn, 11-041 Olsztyn, Poland; marek.drozdowski@uwm.edu.pl

**Keywords:** breast cancer, dietary pattern, Mediterranean diet, hormones, metabolic syndrome

## Abstract

Breast cancer is the most diagnosed cancer in women worldwide. Studies regarding complex breast cancer aetiology are limited and the results are inconclusive. We investigated the associations between dietary patterns (DPs), metabolic-hormone profiles (M-HPs), and breast cancer risk. This case-control study involved 420 women aged 40–79 years from north-eastern Poland, including 190 newly-diagnosed breast cancer cases. The serum concentration of lipid components, glucose, and hormones (oestradiol, progesterone, testosterone, prolactin, cortisol, insulin) was marked in 129 post-menopausal women (82 controls, 47 cases). The food frequency consumption was collected using a validated 62-item food frequency questionnaire. *A posteriori* DPs or M-HPs were derived with a Principal Component Analysis (PCA). Three DPs: ‘Non-Healthy’, ‘Prudent’, and ‘Margarine and Sweetened Dairy’ and two M-HPs: ‘Metabolic-Syndrome’ and ‘High-Hormone’ were identified. The ‘Polish-adapted Mediterranean Diet’ (‘Polish-aMED’) score was calculated. The risk of breast cancer risk was three-times higher (odds ratio (OR): 2.90; 95% confidence interval (95% Cl): 1.62–5.21; *p* < 0.001) in the upper tertile of the ‘Non-Healthy’ pattern (reference: bottom tertile) and five-times higher (OR: 5.34; 95% Cl: 1.84–15.48; *p* < 0.01) in the upper tertile of the ‘High-Hormone’ profile (reference: bottom tertile). There was a positive association of ‘Metabolic-Syndrome’ profile and an inverse association of ‘Polish-aMED’ score with the risk of breast cancer, which disappeared after adjustment for confounders. No significant association between ‘Prudent’ or ‘Margarine and Sweetened Dairy’ DPs and cancer risk was revealed. Concluding, a pro-healthy diet is insufficient to reduce the risk of breast cancer in peri- and postmenopausal women. The findings highlight the harmful effect of the ‘High-Hormone’ profile and the ‘Non-Healthy’ dietary pattern on breast cancer risk. In breast cancer prevention, special attention should be paid to decreasing the adherence to the ‘Non-Healthy’ pattern by reducing the consumption of highly processed food and foods with a high content of sugar and animal fat. There is also a need to monitor the concentration of multiple sex hormones in the context of breast cancer risk.

## 1. Introduction

Cancer is the second-leading cause of death worldwide [1]. Globally, the number of cancer cases and cancer deaths in 2012 was 14 million and eight million, respectively [2]. The most frequently diagnosed cancer and cause of cancer mortality in women worldwide is breast cancer [1]. There were an estimated 1.7 million new breast cancer cases (25.2% of the total cancer cases) and 0.5 million breast cancer deaths (14.7% of the total cancer deaths) in 2012 [1]. In Poland, the number of cancer cases and cancer deaths has increased and was about 156 thousand and 95 thousand, respectively, in 2013 [3]. Breast cancer is the leading cause of cancer incidence (21.9% of the total cancer cases) and the second cause of cancer deaths (13.9% of the total cancer deaths) in Polish women [3,4].

Breast cancer aetiology is composed of many factors, including: age, genetic, reproductive, hormonal, and lifestyle factors, of which diet is of particular interest [1,5]. There are many studies examining the association between dietary factors and breast cancer, and, depending on the strength of conclusions, several grades of evidence have been distinguished [6,7,8,9,10,11,12,13,14]. Strong evidence has been obtained only for alcoholic drink consumption as a convincing and probable factor increasing the risk of post- and premenopausal breast cancer, respectively [14]. There is limited evidence suggesting that non-starchy vegetables (for oestrogen-receptor-negative breast cancer only), foods containing carotenoids, and diets that are high in calcium might decrease the risk of breast cancer, both in pre- and postmenopausal women [14]. Evidence of the impact of the consumption of other foods or nutrients on the risk of breast cancer is too limited to draw any conclusions [14]. This evidence may be limited in many aspects, including the number of studies available, especially a lack of good-quality data, methodological heterogeneity between studies (e.g., adjustment for other confounders), or lack of direct association [14].

An approach that is focused on individual foods or nutrients to assess the association with cancer does not include the complex interactions between the various components of foods [15]. Another approach is to focus on evaluating the diet as a whole, based on dietary patterns (DPs) [15]. Many previous studies have used *a posteriori* analyses, mainly Principal Components Analysis (PCA) or Factor Analysis (FA), to identify dietary patterns [6,7,8,11,12,13]. Less commonly used is an *a priori* approach, like the Mediterranean diet score [16,17,18,19]. There are many studies regarding the association between dietary patterns, including Mediterranean diet and breast cancer risk, however, the findings are inconsistent [6,7,8,11,12,13,16,17]. These discrepancies could result from various study designs, including the race and size of the sample, the composition of the Mediterranean diet, the use of different methods of statistical analysis to identify DPs, or the choice of another set of confounders [20,21,22].

In addition to the dietary factors, breast cancer is linked to metabolic and hormone-related factors, including endogenous sex hormone levels. The contribution of some hormones, especially oestrogens and androgens [23,24,25,26] in hormone-dependent postmenopausal breast cancer pathogenesis has been well established. As for other hormones, such as progesterone or prolactin [27,28], there is not enough evidence for dependence in this regard. Recent data have indicated the possible contribution of cortisol in the pathogenesis of breast cancer [29,30]. Many complex mechanisms indicate the association of many hormones and hormones with metabolic syndrome in the pathogenesis of breast cancer, through interactions in signalling pathways involving oestrogen, insulin, growth factors, and cytokines, especially in the postmenopausal case of hormone-dependent cancers [31,32]. Metabolic syndrome is characterized by at least three of the following metabolic risks: visceral obesity, high blood triglycerides, low high-density lipoprotein cholesterol, high fasting glucose, and hypertension [33]. Several studies have examined the association between metabolic syndrome and its individual components with breast cancer risk [33,34,35,36,37,38]. Due to possible preclinical bias and taking into account the cut-off levels of blood lipid fractions, the results have been inconclusive and they should be interpreted with caution. Therefore, there are no evident associations with breast cancer risk and the underlying mechanisms are not fully understood [35].

Given that breast cancer is an etiologically complex disease, comprised of lifestyle, molecular, and metabolic factors, a transdisciplinary approach is the key to understanding the mechanisms linking diet, metabolic syndrome, and hormones with cancer. Previous studies have examined the association of dietary patterns [6,7,8,9,10,11,12,13,16,17,18,19,20,21,22] or the individual endogenous hormone levels [23,24,25,26,27,28,29,30] or the individual metabolic syndrome components with breast cancer risk [33,34,35,36,37,38]. However, to our knowledge, no studies investigating all of the factors mentioned above in regard to breast cancer have been published to date.

The aim of the study was to assess the associations of dietary patterns, including ‘Polish-adapted Mediterranean Diet’ (‘Polish-aMED’) score and metabolic-hormone profiles (M-HPs) with breast cancer risk in women from north-eastern Poland. Here, we further investigated these observations by also taking into consideration many potential confounders, including hormone receptor status.

## 2. Materials and Methods

### 2.1. Ethical Approval

The study was approved by the Bioethics Committee of the Faculty of Medical Sciences, University of Warmia and Mazury in Olsztyn on 2 October 2013 (resolution no. 29/2013). All of the subjects gave their written informed consent to participate in the study, including to blood sample collection and to use clinical data for research.

### 2.2. Study Design and Sample Characteristics

The present study was conducted in 2014–2017 among adult women from north-eastern Poland. The study protocol with a case-control design was developed. In total, the cancer-control sample involved 420 subjects, aged 40.0–79.9 (mean 59.9 SD 8.6) years.

**Figure 1:**
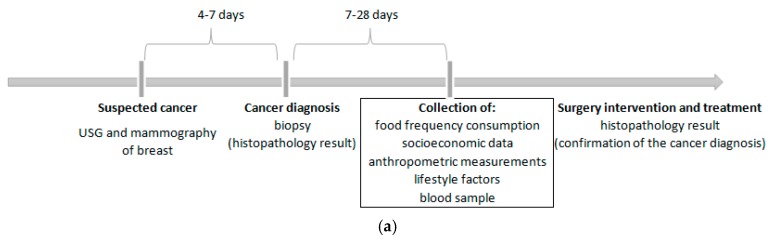

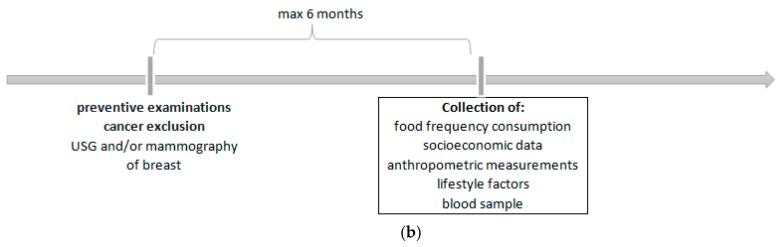
(**a**) Time-based study design—the cancer sample. (**b**) Time-based study design—the control sample. USG—ultrasonography.

The cancer sample involved 190 women with a newly diagnosed (primary) and histologically confirmed breast cancer (invasive or in situ) identified by codes C50.0–50.9 of the International Classification of Diseases for Oncology [39]. The time from cancer diagnosis to case recruitment in the study and data collection ranged from seven days to 28 days (Figure 1a). Breast cancer cases were patients of the surgical oncology ward at the Ministry of Internal Affairs Hospital with the Warmia and Mazury Oncology Centre in Olsztyn. The exclusion criteria of the cancer sample collection were described previously [40]. Briefly, cases diagnosed of other cancer or secondary breast cancer, or with benign changes, after active treatment (e.g., chemotherapy, hormone therapy, radiotherapy) or surgical intervention were not eligible for participation in the study.

**Figure 2:**
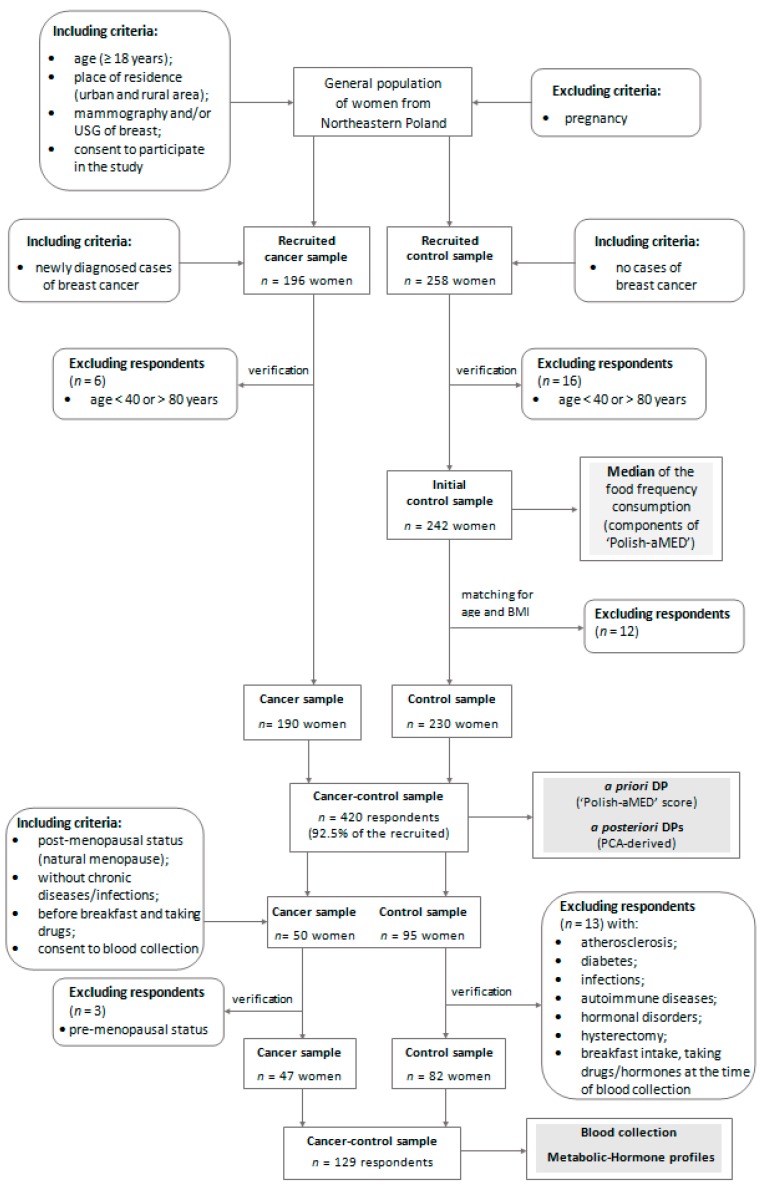
Diagram of the study design and sample collection. BMI—Body Mass Index; USG—ultrasonography; DP—dietary pattern; ‘Polish-aMED’—‘Polish-adapted Mediterranean Diet’; PCA—Principal Component Analysis; The stages of the study are shaded.

The control sample involved 230 women who were excluded from breast cancer based on mammography (MM) and/or ultrasonography (USG) of the breasts. The time from cancer exclusion to subject recruitment in the study and data collected did not exceed six months (Figure 1b). Control subjects did not have any cancer or benign changes in their medical history. The control sample were women who visited the Centre for Prevention and Breast Diagnostics in Olsztyn and other clinics in north-eastern Poland and attended in the prophylactic screening of breast cancer. Details of the study design and the collection of cancer and control samples are shown in Figure 2.

Breast cancer diagnosis and pathologic characteristics of the tumours were confirmed through surgery results and oncology consultant reports. Information that was related to the hormone receptor status was obtained for tumours with available results of the immunohistochemical analyses of the breast cancer tissue in the medical record (Table 1). These data were available for 140 of the 190 breast cancer cases. In the most cases, the ductal tumours (81.4%), with positive oestrogen (ER+) and progesterone receptor status (PR+) and negative human epidermal growth factor receptor 2 (HER2-) of cancer (70.0%; Luminal A) were diagnosed.

### 2.3. Food Frequency Consumption and Polish-Adapted Mediterranean Diet Score

Dietary data were collected using a validated and interviewer-administered version of the 62-item Food Frequency Questionnaire (FFQ-6) [41]. The wide application and validation results of the FFQ-6 were described elsewhere [40]. In brief, respondents provided information about the frequency consumption of 62 food groups at least 12 months prior to participation in the study. The frequency consumption (six categories) was expressed as times/day after assigning the appropriate values as given below in Supplementary Table S1. The consumption frequency of some food groups was summed up to form 21 food groups (Table S1).

The ‘Polish-adapted Mediterranean Diet’ (‘Polish-aMED’) score is a Polish version of the Mediterranean diet (MED) score described by Fung et al. [42]. In developing the ‘Polish-aMED’ score, updated evidence of breast cancer risk factors was considered [14]. The ‘Polish-aMED’ score was calculated based on the qualitative data of the frequency of consumption (times/day) of eight selected dietary items. The set of food items included in the ‘Polish-aMED’ score, and details of its calculation are shown in the Supplementary Table S2 and were described previously [40]. The adherence to the ‘Polish-aMED’ score was expressed in a range from 0 to 8 points and it was considered at three levels established *a priori*: low (0–2 points), average (3–5 points), and high (6–8 points).

### 2.4. Blood Sample Collection and Serum Biomarkers Concentration

We limited the blood sample collection to the selected 129 postmenopausal women aged 45.0–79.9 (mean 62.1 SD 8.2) years, including 47 breast cancer cases and 82 controls. Cancer and control sub-samples were chosen through a convenient and non-random selection, without a significant difference in age or BMI. Blood samples from cases were collected after breast cancer diagnosis and prior to surgery or therapy initiation (Figure 1a). All women were free from acute medical conditions, including diabetes, and had not taken any form of hormonal supplement, including hormone replacement therapy (HRT), for the past year prior to blood sampling and at the time of blood collection. Details of including or excluding blood collection criteria are shown in Figure 2.

At baseline, a 12 mL fasting blood sample was obtained from participants via venipuncture (antecubital venous blood) and was collected in two 6 mL pre-labelled and red top tubes (Clot Activator Tube; BD Vacutainer^®^, Franklin Lakes, NJ, USA) between 8:00 a.m. and 10:00 a.m., all according to standardized procedures. Blood samples were allowed to stand for 30 min at 22 °C, and were then centrifuged at 3000 rpm for 10 min at 22 °C, and the separated serum was then obtained. For all analyses, laboratory staff were blind to the case-control status of samples. All methods were fully automated with automatic calibration and performed at the Laboratory of Biochemical Studies of Nutritional Status in the Department of Human Nutrition at the University of Warmia and Mazury in Olsztyn.

Glucose, triglycerides (TG), total-cholesterol (TC), and high-density lipoprotein cholesterol (HDL-C) concentrations were measured in serum samples using a Cobas Integra 400plus auto-analyser (Roche Diagnostics^®^, Basel, Switzerland), according to the manufacturer’s instructions. Glucose was measured enzymatically with the hexokinase method. Enzymatic-colorimetric tests were used to determine TG, TC, and HDL-C. The low-density lipoprotein cholesterol (LDL-C) concentrations were calculated using the Friedwald’s formula. Serum endogenous hormones: oestradiol, progesterone, prolactin, and testosterone and cortisol and insulin concentrations were measured in serum samples with electrochemiluminescence immunoassays (ECLIA) using an automated immune-analyser Cobas e411 (Roche Diagnostics^®^). The minimum detectable concentrations (MDC) were as follows: glucose 4.32 mg/dL, TG 8.85 mg/dL, TC 3.87 mg/dL, HDL-C 3.0 mg/dL, oestradiol 5.0 pg/mL, progesterone 0.03 ng/mL, prolactin 0.047 ng/mL, testosterone 0.025 ng/mL, insulin 0.2 µU/mL, and cortisol 0.054 µg/dL. Women with hormone concentrations below the MDC (oestradiol, *n* = 66; progesterone, *n* = 39; testosterone, *n* = 10) were assigned the value of the minimum level of detection.

### 2.5. Metabolic Syndrome Components

The definition of metabolic syndrome according to the Expert panel on Detection, Evaluation, and Treatment of High Blood Cholesterol in Adults [43], with a slight modification was used. This definition requires the presence of at least three or more of the following components: waist circumference ≥ 88 cm (measured), glucose ≥ 100 mg/dL, HDL-C < 50 mg/dL, TG ≥ 150 mg/dL, and hypertension (self-declared in our study). Metabolic syndrome components were categorized according to the cut-offs that are mentioned above. For LDL/HDL, log TG/HDL, and non-HDL, the cut-offs were based on the values used in the European Guidelines on cardiovascular disease prevention (3.50, 0.50, and 145 mg/dL, respectively) [44]. Women were categorized as: with and without metabolic syndrome and with 0 metabolic syndrome components, 1–2 components, and 3–5 components. The sum of an individual’s number of metabolic syndrome components was used to create a metabolic syndrome score (MetS; range: 0–5).

### 2.6. Confounders

The potential confounders were selected *a priori* according to current knowledge regarding convincing and probable breast cancer risk factors [14]. The list of confounders included the following and is described in Table S3:

- age;
- BMI;
- socioeconomic status;
- overall physical activity;
- age at menarche;
- menopausal status;
- oral contraceptive use;
- hormone-replacement therapy use;
- number of children;
- smoking status;
- abuse of alcohol;
- vitamin/mineral supplement use;
- family history of breast cancer in first- or second-degree relative; and
- molecular of breast cancer subtypes.

### 2.7. Identification of Dietary Patterns and Metabolic-Hormone Profiles

The Principal Component Analysis (PCA) with varimax rotation was applied to identify dietary patterns (DPs) and metabolic-hormone profiles (M-HPs) [45]. Two separate analyses were executed to identify the PCA-derived DPs or PCA-derived M-HPs. All of the input variables were standardized (to achieve mean equal 0 and standard deviation equal 1) before including them into the PCA. The input variables were: (i) the frequency of consumption of 21 food groups (expressed as times/day) to identify the DPs, and (ii) 11 metabolic syndrome components and serum hormone concentration to identify the M-HPs. In identifying the number of PCA-derived DPs or M-HPs, three criterions were considered: (i) the eigenvalues from the correlation matrix of the standardized variables > 1.0, (ii) the break point identified in the plot of eigenvalues, and (iii) the total variance explained [45]. Rotated factor loadings > |0.30| were considered as significantly contributing to each DP or M-HP, and higher factor loading indicated a stronger association between a food group and the dietary pattern or biomarker and metabolic-hormone profile [17,46]. DPs and M-HPs were labelled according to the highest factor loadings for each of their components. Tertile intervals were calculated for each of the PCA-derived DP M-HPs. For each DP or M-HP, the scores were calculated as a sum of the product of the input variables (frequency of food consumption or the biomarkers values, respectively) and its factor loadings.

### 2.8. Statistical Analysis

Differences in baseline characteristics between cases and controls were verified with a Pearson Chi^2^ test (categorical data) or a Kruskal-Wallis test (continuous data). The association between the frequency consumption of 21 food groups and the ‘Polish-aMED’ score was evaluated using Pearson’s correlation coefficients. The percentage distribution of breast cancer cases was compared by tertiles or levels of DPs and M-HPs using the Pearson Chi^2^ test with Yates’ correction as necessary. All serum biomarker concentrations were log-transformed to normalize their variable distributions and Student’s *t*-test was then used to compare them between cases and controls. Logistic regression analysis was used to assess the associations of DPs or M-HPs with breast cancer risk. The odds ratio (OR) and 95% confidence interval (95% CI) were calculated. The references (OR = 1.00) were the control sample and the bottom tertile or lowest level of each DP or M-HP. Four models were created: unadjusted model, model 1—adjusted for the potential confounders mentioned above, model 2 and model 3—fully-adjusted models for the same confounders included in model 1 and for M-HPs Score or DPs Score, respectively (excluding the modelled variable from confounders set). The level of significance of the odds ratio was verified with the Wald’s test [45]. The statistical analysis was performed using STATISTICA software (version 10.0 PL; StatSoft Inc., Tulsa, OK, USA; StatSoft, Krakow, Poland). A *p*-value < 0.05 was considered to be statistically significant.

## 3. Results

Table 2 shows the baseline characteristics of the cancer and control sample. Most of the participants were postmenopausal (85.2%). When compared to the controls (non-cancer), more cases of breast cancer were slightly older, came from a village or town under 20,000 inhabitants, and had a lower education level and lower socioeconomic status. More cancer cases also declared a family history of breast cancer in a first- or second-degree relative, had earlier age at menarche (<12 years) and menopause (<50 years), were less physically active, were current or former smokers, and abused alcohol. As compared to the controls, there were fewer cases of breast cancer among women who had not had a full-term pregnancy and had used vitamin or mineral supplements within the last 12 months (Table 2).

### 3.1. Food Frequency Consumption and Dietary Patterns

A positive correlation was found between the *a priori* ‘Polish-aMED’ score with the frequency consumption of: nuts and seeds, wholemeal cereals, fruit, legumes, vegetables, fish, and with the ratio of vegetable oils-to-animal fat, and a negative correlation with the frequency consumption of red and processed meats regarding components of the ‘Polish-aMED’ score (Table 3).

Using the *a posteriori* approach, three dietary patterns were identified (Table 3). The ‘Non-Healthy’ DP was loaded heavily by the frequent consumption of refined cereals, red and processed meats, sugar, honey and sweets, potatoes, animal fats, vegetable oils, sweetened beverages, and energy drinks (Table 3). The ‘Prudent’ DP reflected mainly the consumption of fruit, fish, legumes, milk, fermented milk drinks and cheese curd, wholemeal cereals, fruit, vegetable or vegetable-fruit juices, eggs, vegetables, nuts and seeds, vegetable oils, breakfast cereals, and cheese (Table 3). The consumption of other fats (margarine, mayonnaise, dressings), sweetened milk beverages and flavoured cheese curds, white meat, and breakfast cereals contributed heavily to the pattern labelled the ‘Margarine and Sweetened Dairy’ (Table 3). The frequency of consumption of food groups by tertiles of DPs is shown in Supplementary Table S1.

### 3.2. Biomarkers and Metabolic-Hormone Profiles

Using the *a posteriori* approach, two metabolic-hormone profiles were identified (Table 4). The ‘Metabolic-Syndrome’ Profile was positively loaded by the waist circumference, hypertension, and serum concentration of triglycerides, insulin, and glucose and was negatively loaded by the serum HDL-C concentration (Table 4). The ‘High-Hormone’ Profile was positively loaded by the serum concentration of progesterone, oestradiol, testosterone, cortisol, and prolactin (Table 4). The serum hormone concentration and metabolic syndrome components by tertiles of the ‘Metabolic-Syndrome’ and ‘High-Hormone’ Profiles are shown in Supplementary Tables S4 and S5.

### 3.3. Dietary Patterns, Metabolic-Hormone Profiles and Breast Cancer Risk

When compared to the controls, the number of breast cancer cases was lower in the high level of the ‘Polish-aMED’ score (29.5% vs. 38.7%) and higher in the upper tertiles of the ‘Non-Healthy’ DP (46.3% vs. 22.6%), ‘Metabolic-Syndrome’ (44.7% vs. 32.8%) and ‘High-Hormone’ Profiles (57.4% vs. 32.8%; Table 5). There were no significant differences in the number of cases and controls within the tertiles of the ‘Prudent’ or ‘Margarine and Sweetened Dairy’ DPs (Table 5).

As compared to the controls, more cases of breast cancer had elevated levels of metabolic syndrome components: waist circumference ≥ 88 cm (65.9% vs. 53.5%), serum concentration of TG ≥ 150 mg/dL (20.0% vs. 7.4%), and HDL-C < 50 mg/dL (26.0% vs. 9.9%), as well as log of TG/HDL ratio ≥ 0.5 (18.0% vs. 6.2%) and LDL/HDL ratio ≥ 3.5 (12.0 vs. 2.5; Table 5). Breast cancer cases had a higher mean waist circumference (94.0 cm vs. 90.4 cm), serum concentration of oestradiol (22.6 pg/mL vs. 8.8 pg/mL), progesterone (0.29 ng/mL vs. 0.09 ng/mL), prolactin (21.3 ng/mL vs. 10.5 ng/mL), testosterone (0.25 ng/mL vs. 0.17 ng/mL), and TG (122.0 mg/dL vs. 94.6 mg/dL) and log of TG/HDL ratio (2.4 vs 1.5) than controls. On the other hand, TC (205.1 mg/dL vs. 219.1 mg/dL), HDL-C (59.6 mg/dL vs. 71.6 mg/dL), and glucose concentration (92.4 mg/dL vs. 98.0 mg/dL) were lower in cases than in controls (Table 5).

In the upper tertile of the ‘Non-Healthy’ DP, the risk of breast cancer was three-times (OR: 2.90; 95% Cl: 1.62–5.21; *p* < 0.001; fully-adjusted model 2; reference: bottom tertile) and four-times higher (OR: 3.78; 95% Cl: 2.29–6.22; *p* < 0.0001; unadjusted model; reference: bottom tertile). A one-point increment in the ‘Non-Healthy’ DP score increased the risk of cancer by 32% (OR: 1.32; 95% Cl: 1.15–1.51; *p* < 0.0001; fully-adjusted model 2) and 40% (OR: 1.40; 95% Cl: 1.24–1.57; *p* < 0.0001; unadjusted model; Table 6). In the high level (6–8 points) of the ‘Polish-aMED’ score, the risk of breast cancer was lower by 56% (OR: 0.44; 95% Cl: 0.23–0.85; *p* < 0.05; unadjusted model; reference: 0–2 points). The one-point increment in the ‘Polish-aMED’ score decreased the risk of cancer by 14% (OR: 0.86; 95% Cl: 0.77–0.96; *p* < 0.01; unadjusted model). This association disappeared after the adjustment (Table 6). The ‘Prudent’ and ‘Margarine and Sweetened Dairy’ DPs were not significantly associated with the risk of breast cancer (Table 6).

In the upper tertile of the ‘High-Hormone’ Profile, the risk of breast cancer was five-times (OR: 5.34; 95% Cl: 1.84–15.48; *p* < 0.01; fully-adjusted model 3; reference: bottom tertile) and six-times higher (OR: 5.76; 95% Cl: 2.20–15.11; *p* < 0.001; unadjusted model; reference: bottom tertile). A one-point increment in the ‘High-Hormone’ Profile score increased the risk of cancer by 6% (OR: 1.06; 95% Cl: 1.02–1.10; *p* < 0.01; unadjusted model) and 7% (OR: 1.07; 95% Cl: 1.02–1.11; *p* < 0.01; fully-adjusted model 3; Table 6). In the upper tertile of the ‘Metabolic-Syndrome’ Profile, the risk of breast cancer was three-times higher (OR: 3.30; 95% Cl: 1.28–8.49; *p* < 0.05; unadjusted model; reference: bottom tertile). A one-point increment in the ‘Metabolic-Syndrome’ Profile score increased the risk of cancer by 1% (OR: 1.01; 95% Cl: 1.00–1.02; *p* < 0.01; unadjusted model. This association disappeared after the adjustment (Table 6). 

## 4. Discussion

To the authors’ best knowledge, this is the first study regarding the association between metabolic-hormone profile and breast cancer risk, and both the *a priori* Mediterranean diet score and *a posteriori*-derived dietary patterns in this area. The data highlight the harmful effect of the ‘High-Hormone’ profile and the ‘Non-Healthy’ pattern on breast cancer risk in peri- and postmenopausal women from north-eastern Poland. A positive association between the ‘Metabolic-Syndrome’ profile and the risk of cancer was found, but it disappeared after adjustment. In regards to the pro-healthy patterns, there was an inverse association of the ‘Polish-aMED’ score with breast cancer and it disappeared after adjustment, and no significant association was found between the ‘Prudent’ pattern and cancer.

### 4.1. Metabolic-Hormone Profiles and Breast Cancer Risk

The findings provide new insight into the importance of cumulative effect of many hormones in breast cancer aetiology. The ‘High-Hormone’ profile was a set of relatively high serum concentrations of oestradiol, testosterone, prolactin, progesterone, and cortisol. A high adherence to the ‘High-Hormone’ profile was associated with a five-fold increase in breast cancer risk, independently of many potential confounders. This strong association was obtained in the case-control study with a smaller strength of inference and needs to be confirmed in large-scale studies. Since the available studies have been mainly focused on a single hormone in association with breast cancer, it is hard to compare our results with others. Our results are consistent with evidence that elevated serum levels of oestradiol or testosterone are linked to a higher risk of breast cancer in postmenopausal women [24,25,26,47]. The indirect pro-carcinogen effect of the increased testosterone levels results from the fact that androgens are precursors of oestrogens, which stimulate breast epithelial proliferation [23]. The ‘High-Hormone’ profile was positively loaded by serum prolactin and progesterone, which are positively associated with postmenopausal breast cancer in case-control [24,48], prospective [49,50], and cross-sectional studies [51]. Experimental data suggest that prolactin and progesterone are involved in breast cancer aetiology by promoting the proliferation and differentiation of mammary epithelial cells [52], however, the number of studies in this regard is limited and there is insufficient evidence. Recent studies also look at the role of stress-related hormones in the aetiology of cancer, e.g., cortisol [30], which was included into the ‘High-Hormone’ profile. However, data regarding cortisol involvement in breast cancer are limited. Possible molecular mechanisms suggest that chronic psychological stress activates the sympathetic nervous system, leading to damage to DNA structures and it contributes to the initiation and progression of cancer [29].

The study showed that *a posteriori*-derived ‘Metabolic-Syndrome’ profile increased the risk of breast cancer three-times, however, this association disappeared after adjustment. These findings are compatible with data from a recent systematic review [53] and Italian cohort studies [33,37] that *a priori* metabolic syndrome increased the breast cancer risk in postmenopausal women by approx. 50% and almost two-times, respectively. Epidemiologic studies that investigated the associations between individual metabolic syndrome components and breast cancer have reported contradictory results [33,34,35,36,37]. In the present study, the ‘Metabolic-Syndrome’ profile was positively loaded by TG, and accordance with other studies, more cases of breast cancer had an elevated serum level of TG than controls [37,54]. However, in recent meta-analysis [34], case-control [33], and cohort studies [55], no association was found. In turn, the ‘Metabolic-Syndrome’ profile was inversely loaded by HDL-C. Similarly, many studies have reported that low serum HDL-C significantly increased the postmenopausal breast cancer risk [34,35,37,54], although not all studies support these findings [33,36,38]. Indeed, experimental studies have provided evidence that HDL-C could protect against carcinogenesis through antioxidant and anti-inflammatory effects [34]. The current study highlighted the indirect importance of waist circumference as a component of the ‘Metabolic-Syndrome’ profile and measure of visceral obesity—the evidenced risk factor of postmenopausal breast cancer [14,56]. However, in some studies, there was no significant association between waist circumference and breast cancer [33,37,38]. The ‘Metabolic-Syndrome’ profile was positively loaded by hypertension, although, as in Agnoli et al. studies [33,37], no difference was found in hypertension between cases and controls. Nonetheless, in a Kabat et al. study [38], a positive association with breast cancer was seen for diastolic blood pressure. In regards to the glucose and insulin, as a component of the ‘Metabolic-Syndrome’ profile, some data suggest that elevated serum levels of these markers may be considered as predictors for breast cancer [33,38,57], however, there is not enough evidence [37,54,55]. This possible association may be explained by increased insulin-like growth factors and inflammatory cytokines that are induced by hyperinsulinemia and insulin resistance [56].

### 4.2. Dietary Patterns and Breast Cancer Risk

The findings strengthen previous studies [40] and show that a high adherence to the ‘Non-Healthy’ DP increased the risk of breast cancer approx. three-times. These results are consistent with available data. Dietary patterns that are composed of processed meat, fast foods, refined grains, sweets, and sweetened or alcoholic beverages described as ‘Western’ or ‘Drinker’ patterns [6,11,22] were associated with an increase of breast cancer risk from 1.2-times in a French cohort study [11] and meta-analysis [6] to approx. 1.5-times in a Spanish case-control study [22] and an Italian cohort EPIC study [58]. This negative effect could result from elevated consumption of high-processed foods with high energy density and high-glycaemic carbohydrates and animal fat content, which are related to positive energy balance and insulin resistance [59]. Moreover, a high consumption of fried, smoked, or grilled red meat may promote cancerogenesis due to a high content of some mutagenic compounds [59]. However, in some studies, dietary patterns labelled as the ‘Western’ or the ‘Unhealthy’/’Non-healthy’ [6,7], the ‘Meat’ [12], the ‘Meat/Potatoes’ [16], and the ‘High-protein, high-fat’ [8,60] were not significantly associated with breast cancer risk.

A high adherence to the ’Polish-aMED’ score reduced the breast cancer risk by 56%, although this beneficial effect disappeared after adjustment. This weak association could result from Poland not belonging to the countries of the Mediterranean region and the Polish diet being unlike the traditional Mediterranean diet. Similarly, in British [10], Dutch [61], Swedish [17], and American [13] cohort studies, no significant association was found between a Mediterranean diet and total breast cancer risk. In contrast, a strong beneficial effect of the ’Polish-aMED’ score has been reported in our recent study [40]. However, this previous analysis was based on pooled data from two case-control samples consisting of men and women, including cases of breast and lung cancer and therefore also different confounders compared to the current study [40]. In addition, data from the Mediterranean region highlight the decrease in breast cancer risk with the high adherence to the Mediterranean pattern by 6% in the EPIC study [18] and updated meta-analysis [21], through 19% in the Italian case-control study [62], and by 44% in a Spanish case-control study [22]. This protective anti-cancer effect of the traditional plant-based Mediterranean diet could result from many bioactive compounds [20]. The discrepancies in the results of the mentioned studies could be explained by a various set of foods items in the Mediterranean pattern composition [21].

In the present study, no associations found between the ‘Prudent’ DP and breast cancer risk is consistent with the results from the authors’ previous study [40]. This pattern comprised of both beneficial dietary items (e.g., vegetables or wholemeal cereals) and those that are considered non-healthy when eaten in greater amounts (e.g., breakfast cereals, eggs, cheese). Thus, in the final result, the ‘Prudent’ DP may have a neutral character in relation to cancer incidence. In accordance with the current results, no significant association with the breast cancer was found for the ‘Cereals/Milk/Dairy’ DP [16], ‘Vegetarian’ DP [63], and the ‘Prudent’ DP composed of fruits, vegetables, whole grains, low-fat dairy, and juices [10,22]. However, in some studies, the ‘Prudent’/’Healthy’ DPs that also included fish and poultry [6,59] or were composed of fruits and vegetables only, such as the ‘Plant-based’ [8], ‘Vegetables’ [58], and ‘Fruit and Salad’ DPs [12], decreased the risk of breast cancer by 11–52%. This anti-cancer effect probably results from a high-quality diet, which is rich in bioactive compounds, including vitamins, minerals, fibre, phenolics, polyunsaturated fatty acids, and specific peptides.

### 4.3. Strengths and Limitations

A unique strength of this study is the novel approach in investigating the breast cancer aetiology by taking into account both many hormones and metabolic components as overall metabolic-hormone profiles. This holistic approach obtained results regarding the combined effects of these biomarkers on breast cancer risk. Furthermore, we have included a number of potential confounders in the fully adjusted model, including socioeconomic, reproduction, lifestyle, clinical factors, and dietary pattern scores. Another strength of the study broadly assesses the respondents’ diets as a whole by identifying dietary patterns using two methods (*a priori* and *a posteriori*) [15,46]. To collect dietary data, a validated interviewer-administrated FFQ was used [41]. Lastly, all measurements, including the concentration of biomarkers, were made just after the cases’ breast cancer diagnoses, prior to treatment or surgery, to avoid the impact of mechanical or psychological stress and the disease itself to prevent false positive results. The concentration of many markers has a strong circadian rhythm, increasing after a noontime meal [49]. Thus, the fasting blood collection was used to minimize misclassification.

There are some study limitations, including its case-control design and sampling bias. A non-random sample selection may reduce the external validity of the study. The close matching of the controls with cases may cause an association existing in real life to be overestimated [64]. However, matching by age and BMI was necessary to reduce the variability of the basic input data in our study. The second limitation is a lack of quantitative data of food consumption and nutrient intake, although in the disease’s associations the usual food consumption needs to be considered, not single diet components [15]. Another limitation of the study was that a single blood draw for each subject was used for the measurement of biomarkers levels, and may not be representative in the long-term. However, the Nurses’ Health Study has found the adequate-to-high internal correlation for most sex hormones measured up to three years apart on the same women [51]. In our study, the analysis of the association of metabolic-hormone profiles with breast cancer risk was restricted to postmenopausal women. These results cannot be directly interpreted for premenopausal women due to the fluctuation changes of the sex hormone levels in the menstrual cycle [49]. Moreover, premenopausal levels of oestradiol or progesterone may have little or no predictive value for breast cancer that is diagnosed after menopause, where the levels of these hormones are relative lower [27]. Therefore, the association between hormones levels and breast cancer risk in premenopausal women remains unclear [27]. Finally, epidemiologic studies suggest that endogenous sex hormones levels are linked specifically with hormone-sensitive tumours [51]. Given the relatively small sample size and available data of molecular subtypes of breast cancer for not all cases, we were not able to perform statistical analyses stratified by the hormone subtype of breast cancer. Nonetheless, the receptor status was included as a confounder in the adjusted regression analysis model.

## 5. Conclusions

The study revealed that pro-healthy diet is insufficient to reduce the risk of breast cancer in peri- and postmenopausal women. These findings provide interesting insights into the strong harmful effect of the high adherence to the ‘High-Hormone’ profile and the ‘Non-Healthy’ dietary pattern on breast cancer risk, independently of many potential confounders, among women from north-eastern Poland.

The results may improve the understanding of the complex aetiology of breast cancer, related to diet, hormone, and metabolic association and may prove useful in establishing primary preventive strategies. In the prevention of breast cancer, special attention should be paid to decreasing adherence to the ‘Non-Healthy’ pattern by reducing the consumption of highly processed food, foods with a high content of sugar and animal fat. There is also a need to monitor the concentration of multiple sex hormones in the context of breast cancer. Further, large prospective studies are needed to confirm the results on the role of diet, hormones, and metabolic syndrome in the aetiology of breast cancer, stratified by menopausal and hormone receptor status.

## Figures and Tables

**Table 1:** Histologic and molecular subtypes of breast cancer sub-sample.

Variable	Cancer Sub-Sample
*Sample size*	140
	*N* (%)
*Breast*	
right	71 (50.7)
left	69 (49.3)
*Histologic BC subtypes*	
ductal carcinoma	114 (81.4)
lobular carcinoma	15 (10.7)
mixed carcinoma	11 (7.9)
*Molecular BC subtypes—hormone receptor status*	
ER-negative tumours	25 (17.9)
ER-positive tumours	115 (82.1)
PR-negative tumours	43 (30.7)
PR-positive tumours	97 (69.3)
HER2-negative tumours	115 (82.1)
HER2-positive tumours	25 (17.9)
ER−, PR−	22 (15.7)
ER−, PR+	3 (2.1)
ER+, PR−	21 (15.0)
ER+, PR+	94 (67.1)
Triple negative (ER−, PR−, HER2−)	17 (12.1)
ER−, PR−, HER2+ subtype	5 (3.6)
Luminal A (ER+ and or PR+, HER2−)	98 (70.0)
Luminal B (ER+ and or PR+, HER2+)	20 (14.3)

BC—breast cancer; ER—oestrogen receptor status of tumour; PR—progesterone receptor status of tumour; HER2—human epidermal growth factor receptor 2; %—sample percentage.

**Table 2:** Cancer and control sample characteristics (%).

Variable	Cancer-Control Sample	Cancer Sample	Control Sample	*p*-Value
*Sample size*	420	190	230	
*Age* (years *)	59.9 (8.6)	60.9 (9.7)	59.1 (7.4)	0.0210
40.0–49.9	15.5	18.4	13.0	
50.0–59.9	30.0	23.7 ^a^	35.2 ^a^	0.0119
60.0–69.9	42.6	42.1	43.0	
70.0–79.9	11.9	15.8 ^b^	8.7 ^b^	
*BMI* (kg/m^2^ *)	27.9 (5.0)	28.3 (4.8)	27.6 (5.0)	ns
<18.5	0.7	0.5	0.9	
18.5–24.9	29.2	25.0	32.6	ns
25.0–29.9	39.0	40.4	37.8	
≥30.0	31.1	34.0	28.7	
*Place of residence*				
village	28.1	31.1	25.7	
town (<20,000 inhabitants)	15.2	24.2 ^a^	7.8 ^a^	<0.0001
town (20–100,000 inhabitants)	20.5	19.5	21.3	
city (>100,000 inhabitants)	36.2	25.3 ^b^	45.2 ^b^	
*Education level*				
primary	13.6	22.1 ^a^	6.5 ^a^	
secondary	58.3	61.6	55.7	<0.0001
higher	28.1	16.3 ^b^	37.8 ^b^	
*Economic situation*				
below the average	16.0	18.9	13.5	
average	71.2	70.5	71.7	ns
above average	12.9	10.5	14.8	
*Household situation*				
we live poorly	0.2	0.5	0.0	
we live very thriftily	16.9	19.5	14.8	
we live thriftily	56.0	58.4	53.9	ns
we live well	24.8	20.0 ^a^	28.7 ^a^	
we live very well	2.1	1.6	2.6	
*Socioeconomic status* (SES Index *)	9.9 (2.1)	9.3 (2.1)	10.4 (2.0)	<0.0001
low	41.0	53.2 ^a^	30.9 ^a^	
average	36.7	33.2	39.6	<0.0001
high	22.4	13.7 ^b^	29.6 ^b^	
*Physical activity at work*				
low	54.0	66.3 ^a^	43.9 ^a^	
moderate	32.6	23.2 ^b^	40.4 ^b^	<0.0001
high	13.3	10.5	15.7	
*Physical activity at leisure time*				
low	22.6	30.0 ^a^	16.5 ^a^	
moderate	64.3	63.2	65.2	<0.0001
high	13.1	6.8 ^b^	18.3 ^b^	
*Overall physical activity*				
low	52.9	67.9 ^a^	40.4 ^a^	
moderate	44.0	30.5 ^b^	55.2 ^b^	<0.0001
high	3.1	1.6	4.3	
*Sleep* (h)				
<6	19.0	20.0	18.3	
6–8	69.0	67.4	70.4	ns
>8	11.9	12.6	11.3	
*Smokers*	53.1	57.9	49.1	ns
*Current smokers*	21.0	26.8	16.1	0.0070
*Number of cigarettes smoked/day* (current smokers)				
<10	47.2	40.4 ^a^	56.8 ^a^	
11–20	38.2	40.4	35.1	ns
21–40	13.5	17.3 ^b^	8.1 ^b^	
>40	1.1	1.9 ^c^	0.0 ^c^	
*Former smokers* (years)	51.0	56.3	46.5	0.0457
<5	18.7	17.8	19.6	
5–10	13.6	12.1	15.0	ns
>10	67.8	70.1	65.4	
*Number of cigarettes smoked/day* (former smokers)				
<10	42.5	36.4 ^a^	48.6 ^a^	
11–20	42.1	42.1	42.1	ns
21–40	14.5	20.6 ^b^	8.4 ^b^	
>40	0.9	0.9	0.9	
*Passive smokers*	56.4	54.7	57.8	ns
*Current passive-smokers*	16.4	16.8	16.1	ns
(h/day *)	3.3 (3.3)	2.8 (1.9)	3.6 (4.1)	ns
Former passive-smokers	52.6	51.6	53.5	ns
(years *)	19.2 (11.8)	19.4 (12.1)	19.1 (11.6)	ns
(h/day *)	4.5 (2.8)	4.4 (3.0)	4.5 (2.6)	ns
*Abuse of alcohol*	4.0	7.4	1.3	0.0017
*Age at menarche* (years)				
<12	12.1	16.3 ^a^	8.7 ^a^	
12–14.9	63.3	63.2	63.5	0.0268
≥15	24.5	20.5	27.8	
*Menopausal status*				
pre-menopausal	14.8	15.3	14.3	ns
post-menopausal	85.2	84.7	85.7	
*Age at menopause* (years)				
<50	40.8	47.2 ^a^	35.5 ^a^	0.0254
≥50	59.2	52.8 ^b^	64.5 ^b^	
*Number of full-term pregnancies*				
0	12.1	7.9 ^a^	15.7 ^a^	
1–2	61.7	61.6	61.7	0.0219
≥3	26.2	30.5	22.6	
*Age at first full-term pregnancy* (years)				
<20.0	14.1	16.6	11.9	
20.0–29.9	78.9	77.7	79.9	ns
≥30.0	7.0	5.7	8.2	
*Age at last full-term pregnancy* (years)				
<20.0	1.5	2.5	0.6	
20.0–29.9	57.4	60.5	54.5	ns
≥30.0	41.0	37.0	44.9	
*Vitamin/mineral supplements use*	38.6	31.1	44.8	0.0040
*Oral contraceptive use (ever)*	20.2	17.9	22.2	ns
(years *)	4.1 (4.3)	4.7 (4.9)	3.7 (3.8)	ns
*Hormone-replacement therapy use (ever)*	16.7	15.3	17.8	ns
(years *)	4.8 (4.7)	5.4 (5.6)	4.4 (3.9)	ns
*Breastfeeding*^#^ (months)				
≤6	52.2	55.4	49.2	
7–12	24.5	20.0 ^a^	28.5 ^a^	ns
13–24	15.8	17.7	14.0	
>24	7.6	6.9	8.3	
*Family history of BC* ^$^	19.3	24.7	14.8	0.0349
*Diagnosed chronic diseases*	56.9	53.7	59.6	ns
*Surgical interventions*	61.0	64.2	58.3	ns

BMI—body mass index, data missing (*n* = 2); SES—socioeconomic status; BC—breast cancer; ^#^ breastfeeding duration was the number of months of the longest reported breastfeeding; ^$^ in first- or second-degree relative; %—sample percentage; * mean and standard deviation (SD); *p*-value—level of significance verified with chi^2^ test (categorical variables) or Kruskal-Wallis’ test (continuous variables); a–a, b–b, c–c—statistically significant differences between cancers and controls, *p* < 0.05; ns—statistically insignificant.

**Table 3:** Factor loadings for food groups in PCA-derived dietary patterns and the Pearson’s correlation coefficients for ‘Polish-aMED’ score among peri- and post-menopausal women (*n* = 420).

Food Groups	PCA-Derived Dietary Patterns			‘Polish-aMED’ Score
	‘Non-Healthy’	‘Prudent’	‘Margarine and Sweetened Dairy’	
Refined cereals	**0.67**	−0.25	0.12	−0.41 *
Red and processed meats	**0.63**	0.06	0.07	−0.34 *
Sugar, honey and sweets	**0.57**	0.13	0.04	−0.14 *
Potatoes	**0.55**	−0.04	−0.02	−0.20 *
Animal fats	**0.49**	0.12	**−0.66**	−0.31 *
Vegetable oils (including olive oil)	**0.34**	**0.36**	0.02	0.14 *
Sweetened beverages and energy drinks	**0.32**	0.13	0.18	0.01
Fruit	−0.06	**0.55**	−0.05	0.38 *
Fish	−0.07	**0.49**	0.09	0.33 *
Legumes	0.00	**0.48**	0.19	0.36 *
Milk, fermented milk drinks and cheese curd	−0.05	**0.48**	0.13	0.22 *
Wholemeal cereals	**−0.45**	**0.47**	−0.01	0.43 *
Fruit, vegetable, vegetable-fruit juices	0.16	**0.45**	0.00	0.11 *
Eggs	0.20	**0.44**	−0.10	0.10 *
Vegetables	0.00	**0.42**	−0.17	0.34 *
Nuts and seeds	**−0.39**	**0.42**	−0.12	0.46 *
Breakfast cereals	0.04	**0.35**	**0.31**	0.14 *
Cheese	0.23	**0.34**	0.10	0.02
Other fats (margarine, mayonnaise, dressings)	0.17	−0.12	**0.80**	−0.05
Sweetened milk beverages and flavoured cheese	0.29	0.28	**0.36**	−0.06
White meat	0.22	0.08	**0.31**	−0.03
Ratio of vegetable oils to animal fat	NA	NA	NA	0.38 *
Share in explaining the variance (%)	13	12	7	NA

‘Polish-aMED’—‘Polish-adapted Mediterranean Diet’ (range of points: 0–8); PCA—Principal Component Analysis; NA—not applied; bolded values are marked for the main components of PCA-derived dietary patterns with absolute loadings ≥ 0.3 and for eight components of the ‘Polish-aMED’ score; * *p* < 0.05, test of significance for Pearson’s correlation coefficients.

**Table 4:** Factor loadings for metabolic syndrome components and serum hormone concentration in PCA-derived profiles among post-menopausal women (*n* = 129).

Biomarkers	Metabolic-Hormone Profiles	
	‘Metabolic-Syndrome’	‘High-Hormone’
HDL-cholesterol	**−0.76**	−0.27
Waist circumference	**0.72**	−0.05
Hypertension	**0.58**	0.05
Triglycerides	**0.56**	0.01
Insulin	**0.54**	−0.13
Glucose	**0.38**	−0.11
Progesterone	−0.17	**0.83**
Oestradiol	−0.07	**0.77**
Testosterone	0.13	**0.58**
Cortisol	−0.15	**0.44**
Prolactin	0.09	**0.38**
Share in explaining the variance (%)	21	19

HDL—high-density lipoprotein; PCA—Principal Component Analysis; bolded values are marked for the main components of PCA-derived profiles with absolute loadings ≥ 0.3.

**Table 5:** ‘Polish-aMED’ score, Principal Component Analysis (PCA)-derived dietary patterns and metabolic-hormone profiles and their components in association with breast cancer (%).

Variable	Cancer-Control Sample	Cancer Sample	Control Sample	*p*-Value
*Sample size*	420	190	230	
*‘Polish-aMED’ score* (points) *	4.7 (1.8)	4.4 (1.8)	4.9 (1.7)	0.0081
*levels* (points)				
low (0–2)	12.1	15.8 ^a^	9.1 ^a^	
average (3–5)	53.3	54.7	52.2	0.0390
high (6–8)	34.5	29.5 ^b^	38.7 ^b^	
*‘Non-Healthy’ DP*				
*score* *	3.5 (1.8)	4.1 (1.9)	3.1 (1.6)	<0.0001
*tertiles*				
bottom	33.1	22.6 ^a^	41.7 ^a^	
middle	33.6	31.1	35.7	<0.0001
upper	33.3	46.3 ^b^	22.6 ^b^	
*‘Prudent’ DP*				
*score* *	3.4 (1.2)	3.3 (1.2)	3.5 (1.3)	ns
*tertiles*				
bottom	33.1	33.2	33.0	
middle	33.3	33.7	33.0	ns
upper	33.6	33.2	33.9	
*‘Margarine and Sweetened Dairy’ DP*				
*score* *	0.1 (1.0)	0.2 (1.0)	0.1 (1.0)	ns
*tertiles*				
bottom	33.3	33.7	33.0	
middle	33.1	29.5	36.1	ns
upper	33.6	36.8	30.9	
*Sample size*	129	47	82	
*‘Metabolic-Syndrome’ Profile*				
*score* *	111.4 (42.0)	125.6 (49.0)	103.1 (35.1)	0.0032
tertiles				
bottom	40.7	21.3 ^a^	33.6 ^a^	
middle	33.3	34.0	33.6	0.0379
upper	25.9	44.7 ^b^	32.8 ^b^	
*‘High-Hormone’ Profile*				
*score* *	−10.2 (33.5)	2.0 (52.1)	−17.3 (9.4)	0.0015
*tertiles*				
bottom	39.5	21.3 ^a^	32.8 ^a^	
middle	42.0	21.3 ^b^	34.4 ^b^	<0.0001
upper	18.5	57.4 ^c^	32.8 ^c^	
*Hormones*				
oestradiol (pg/mL) *	13.9 (39.9)	22.6 (63.8)	8.8 (11.1)	0.0399
progesterone (ng/mL) *	0.16 (0.42)	0.29 (0.68)	0.09 (0.04)	0.0032
prolactin (ng/mL) *	14.5 (20.5)	21.3 (32.7)	10.5 (3.6)	0.0303
testosterone (ng/mL) *	0.20 (0.12)	0.25 (0.14)	0.17 (0.10)	0.0088
*Sample size*	132	50	82	
cortisol (μg/dL) *	15.6 (7.0)	16.9 (9.1)	14.8 (5.1)	ns
insulin (μU/mL) *	10.2 (7.4)	11.2 (9.7)	9.6 (5.5)	ns
*Metabolic Syndrome Biomarkers*				
triglycerides (mg/dL) *	105.0 (50.0)	122.0 (65.0)	94.6 (34.4)	0.0040
<150	87.8	80.0 ^a^	92.6 ^a^	0.0325
≥150	12.2	20.0 ^b^	7.4 ^b^	
HDL-cholesterol (mg/dL) *	67.0 (17.6)	59.6 (15.8)	71.6 (17.2)	<0.0001
≥50	84.0	74.0 ^a^	90.1 ^a^	0.0145
<50	16.0	26.0 ^b^	9.9 ^b^	
glucose (mg/dL) *	95.9 (11.1)	92.4 (13.7)	98.0 (8.5)	0.0012
<100	67.2	76.0 ^a^	61.7 ^a^	ns
≥100	32.8	24.0 ^b^	38.3 ^b^	
hypertension (self-reported) ^c^	27.1	31.1	23.9	ns
waist circumference (cm) *^d^	92.0 (13.2)	94.0 (13.7)	90.4 (12.6)	0.0062
<88	41.1	34.1 ^a^	46.5 ^a^	0.0112
≥88	58.9	65.9 ^b^	53.5 ^b^	
Metabolic Syndrome Score *	1.4 (1.3)	1.6 (1.3)	1.3 (1.3)	ns
*No. of Metabolic Syndrome Biomarkers*				
0	29.8	22.0 ^a^	34.6 ^a^	
1–2	51.1	56.0	48.1	ns
3–5	19.1	22.0	17.3	
without metabolic syndrome (0–2)	80.9	78.0	82.7	ns
metabolic syndrome (3–5)	19.1	22.0	17.3	
total cholesterol (mg/dL) *	213.8 (41.6)	205.1 (46.4)	219.1 (37.6)	0.0397
LDL-cholesterol (mg/dL) *	126.8 (36.7)	121.8 (40.9)	129.8 (33.7)	ns
log TG/HDL *	1.8 (1.4)	2.4 (1.9)	1.5 (0.8)	0.0001
<0.50	89.3	82.0 ^a^	93.8 ^a^	0.0333
≥0.50	10.7	18.0 ^b^	6.2 ^b^	
LDL/HDL *	2.0 (0.9)	2.2 (1.1)	1.9 (0.6)	ns
<3.50	93.9	88.0 ^a^	97.5 ^a^	0.0269
≥3.50	6.1	12.0 ^b^	2.5 ^b^	
non-HDL (mg/dL) *	146.8 (38.6)	145.5 (46.2)	147.5 (33.3)	ns
<145	48.1	48.0	48.1	ns
≥145	51.9	52.0	51.9	

‘Polish-aMED’—‘Polish-adapted Mediterranean Diet’ (range of points: 0–8); DP—dietary pattern; HDL—high-density lipoprotein; LDL—low-density lipoprotein; TG—triglycerides; %—sample percentage; * mean and standard deviation (SD); ^c^ data for *n* = 420; ^d^ data for *n* = 409; *p*-value—level of significance assessed by chi^2^ test (categorical variables) or Kruskal-Wallis’ test (continuous variables) or Student’s *t*-test (for log-transformed serum biomarkers concentration); a–a, b–b—statistically significant differences between the pairs of cancer and control sample, *p* < 0.05; ns—statistically insignificant.

**Table 6:** Odds ratios (ORs) and 95% confidence interval (95% CI) of breast cancer by adherence to the dietary patterns (*n* = 420) and metabolic-hormone profiles (*n* = 129).

Dietary Patterns/Metabolic-Hormone Profiles	Tertiles/Levels	Sample Size	Breast Cancer							
			Unadjusted Model		Model 1		Model 2		Model 3	
			ORs	95% CI	ORs	95% CI	ORs	95% CI	ORs	95% CI
‘Polish-aMED’	low (0–2 points; ref.)	51	1.00 (referent)		1.00 (referent)		1.00 (referent)		1.00 (referent)	
	average (3–5 points)	224	0.61	0.33; 1.13	0.52	0.26; 1.01	0.53	0.27; 1.05	NA	
	high (6–8 points)	145	0.44 *	0.23; 0.85	0.54	0.26; 1.10	0.52	0.25; 1.07	NA	
	1-point increase ^#^		0.86 **	0.77; 0.96	0.93	0.82; 1.05	0.92	0.81; 1.05	NA	
‘Non-Healthy’	bottom (ref.)	139	1.00 (referent)		1.00 (referent)		1.00 (referent)		1.00 (referent)	
	middle	141	1.61	0.98; 2.63	1.23	0.72; 2.11	1.13	0.65; 1.95	NA	
	upper	140	3.78 ****	2.29; 6.22	3.08 ***	1.74; 5.46	2.90 ***	1.62; 5.21	NA	
	1-point increase ^#^		1.40 ****	1.24; 1.57	1.34 ****	1.17; 1.53	1.32 ****	1.15; 1.51	NA	
‘Prudent’	bottom (ref.)	139	1.00 (referent)		1.00 (referent)		1.00 (referent)		1.00 (referent)	
	middle	140	1.02	0.61; 1.68	1.23	0.73; 2.08	1.27	0.74; 2.17	NA	
	upper	141	0.97	0.61; 1.55	1.47	0.85; 2.57	1.46	0.83; 2.58	NA	
	1-point increase ^#^		0.93	0.80; 1.09	1.05	0.87; 1.26	1.04	0.86; 1.26	NA	
‘Margarine and Sweetened Dairy’	bottom (ref.)	140	1.00 (referent)		1.00 (referent)		1.00 (referent)		1.00 (referent)	
	middle	139	0.80	0.50; 1.30	0.98	0.57; 1.70	0.93	0.53; 1.61	NA	
	upper	141	1.17	0.73; 1.88	1.29	0.76; 2.20	1.15	0.64; 2.06	NA	
	1-point increase ^#^		1.07	0.88; 1.30	1.05	0.84; 1.30	0.99	0.79; 1.24	NA	
‘Metabolic-Syndrome’	bottom (ref.)	43	1.00 (referent)		1.00 (referent)		1.00 (referent)		1.00 (referent)	
	middle	43	1.96	0.75; 5.07	1.65	0.60; 4.53	NA		1.59	0.55; 4.54
	upper	43	3.30 *	1.28; 8.49	1.97	0.68; 5.75	NA		1.61	0.53; 4.89
	1-point increase ^#^		1.01 **	1.00; 1.02	1.01	1.00; 1.02	NA		1.01	1.00; 1.02
‘High-Hormone’	bottom (ref.)	42	1.00 (referent)		1.00 (referent)		1.00 (referent)		1.00 (referent)	
	middle	44	0.94	0.34; 2.60	1.05	0.39; 2.79	NA		0.98	0.34; 2.79
	upper	43	5.76 ***	2.20; 15.11	5.05 **	1.80; 14.19	NA		5.34 **	1.84; 15.48
	1-point increase ^#^		1.06 **	1.02; 1.10	1.07 **	1.03; 1.11	NA		1.07 **	1.02; 1.11

‘Polish-aMED’—‘Polish-adapted Mediterranean Diet’ (range of points: 0–8); ^#^ for dietary pattern or metabolic-hormone profile score; NA—not applied; Model 1—age (categorical variable), BMI (≤24.9, 25.0–29.9, ≥30.0 kg/m^2^), socioeconomic status (low, average, high), overall physical activity (low, moderate, high), smoking status (non-smoker, smoker), abuse of alcohol (no, yes), age at menarche (<12, 12–14.9, ≥15 years), menopausal status (pre-, postmenopausal), number of children (0, 1–2, ≥3), oral contraceptive use (no, yes), hormone-replacement therapy use (no, yes), family history of breast cancer in first- or second-degree relative (no, I don’t know, yes), vitamin/mineral supplements use (no, yes) and molecular of breast cancer subtypes (triple negative, ER−, PR−, HER2+ subtype, luminal A, luminal B) adjusted model; Model 2—model was adjusted for the same variables included in model 1 plus ‘Metabolic-Syndrome’ and ‘High-Hormone’ Profiles Score (fully-adjusted model), excluding the modelled variable from confounders set, respectively; Model 3—model was adjusted for the same variables included in model 1 plus ‘Polish-aMED’ and PCA-driven DPs Score (fully-adjusted model), excluding the modelled variable from confounders set, respectively; 95% CI—95% confidence interval; *p*-value—the level of significance verified with Wald’s test; * *p* < 0.05, ** *p* < 0.01, *** *p* < 0.001, **** *p* < 0.0001.

## Supplementary Materials

The following are available online at http://www.mdpi.com/2072-6643/10/12/2013/s1. Table S1: The mean (95% CI) of the frequency of food consumption by dietary patterns for cancer-control sample (times/day), Table S2: Description of food groups for the ‘Polish-adapted Mediterranean Diet’ score (0–8 points) calculation—data for the Initial control sample (*n* = 242), Table S3: Confounders in the case-control study regarding association of dietary patterns and metabolic-hormone profiles with breast cancer risk, Table S4: ‘High-Hormone’ profile, serum hormone concentration and metabolic syndrome components by ‘Metabolic-Syndrome’ profile (%), Table S5: ‘Metabolic-Syndrome’ profile, metabolic syndrome components and serum hormone concentration by ‘High-Hormone’ profile (%).

## References

[1] (2015). World Cancer Report 2014.

[2] Ferlay J., Soerjomataram I., Ervik M., Dikshit R., Eser S., Mathers C., Rebelo M., Parkin D.M., Forman D., Bray F. (2013). GLOBOCAN 2012 v1.0, Cancer Incidence and Mortality Worldwide: IARC Cancer Base No. 11.

[3] Krajowy Rejestr Nowotworów, Centrum Onkologii—Instytut im. Marii Skłodowskiej—Curie (Polish National Cancer Registry, Oncology Centre Institute of M. Sklodowska-Curie). http://onkologia.org.pl/k/epidemiologia/.

[4] (2014). World Health Organization—Cancer Country Profiles. http://www.who.int/cancer/country-profiles/pol_en.pdf?ua=1.

[5] Vineis P., Wild C.P. (2014). Global cancer patterns: Causes and prevention. Lancet.

[6] Brennan S.F., Cantwell M.M., Cardwell C.R., Velentzis L.S., Woodside J.V. (2010). Dietary patterns and breast cancer risk: A systematic review and meta-analysis. Am. J. Clin. Nutr..

[7] Buck K., Vrieling A., Flesch-Janys D., Chang-Claude J. (2011). Dietary patterns and the risk of postmenopausal breast cancer in a German case-control study. Cancer Causes Control.

[8] Link L.B., Canchola A.J., Bernstein L., Clarke C.A., Stram D.O., Ursin G., Horn-Ross P.L. (2013). Dietary patterns and breast cancer risk in the California Teachers Study cohort. Am. J. Clin. Nutr..

[9] Bessaoud F., Tretarre B., Daures J.P., Gerber M. (2012). Identification of dietary patterns using two statistical approaches and their association with breast cancer risk: A case-control study in southern France. Ann. Epidemiol..

[10] Pot G.K., Stephen A.M., Dahm C.C., Key T.J., Cairns B.J., Burley V.J., Cade J.E., Greenwood D.C., Keogh R.H., Bhaniani A. (2014). Dietary patterns derived with multiple methods from food diaries and breast cancer risk in the UK Dietary Cohort Consortium. Eur. J. Clin. Nutr..

[11] Cottet V., Touvier M., Fournier A., Touillaud M.S., Lafay L., Clavel-Chapelon F., Boutron-Ruaulty M. (2009). Postmenopausal Breast Cancer Risk and Dietary Patterns in the E3N-EPIC Prospective Cohort Study. Am. J. Epidemiol..

[12] Baglietto L., Krishnan K., Severi G., Hodge A., Brinkman M., English D.R., McLean C., Hopper J.L., Giles G.G. (2011). Dietary patterns and risk of breast cancer. Br. J. Cancer.

[13] Hirko K.A., Willett W.C., Hankinson S.E., Rosner B.A., Beck A.H., Tamimi R.M., Eliassen A.H. (2016). Healthy dietary patterns and risk of breast cancer by molecular subtype. Breast Cancer Res. Treat..

[14] World Cancer Research Fund/American Institute for Cancer Research (2018). Continuous Update Project Expert Report 2018. Diet, Nutrition, Physical Activity, and Breast Cancer.

[15] Wirfält E., Drake I., Wallström P. (2013). What do review papers conclude about food and dietary patterns?. Food Nutr. Res..

[16] Demetriou C.A., Hadjisavvas A., Loizidou M.A., Loucaides G., Neophytou I., Sieri S., Kakouri E., Middleton N., Vineis P., Kyriacou K. (2012). The Mediterranean dietary pattern and breast cancer risk in Greek-Cypriot women: A case control study. BMC Cancer.

[17] Couto E., Sandin S., LÖf M., Ursin G., Adami H.O., Weiderpass E. (2013). Mediterranean dietary pattern and risk of breast cancer. PLoS ONE.

[18] Buckland G., Travier N., Cottet V., Gonzalez C.A., Lujan-Barroso L., Agudo A., Trichopoulou A., Lagiou P., Trichopoulos D., Peeters P.H. (2013). Adherence to the Mediterranean diet and risk of breast cancer in the European Prospective Investigation into Cancer and Nutrition cohort study. Int. J. Cancer.

[19] Voevodina O., Billich C., Arand B., Nagel G. (2013). Association of Mediterranean diet, dietary supplements and alcohol consumption with breast density among women in South Germany: A cross-sectional study. BMC Public Health.

[20] Sofi F., Macchi C., Abbate R., Gensini G.F., Casini A. (2014). Mediterranean diet and health status: An updated meta-analysis and a proposal for a literature-based adherence score. Public Health Nutr..

[21] Schwingshackl L., Schwedhelm C., Galbete C., Hoffmann G. (2017). Adherence to Mediterranean diet and risk of cancer: An updated systematic review and meta-analysis. Nutrients.

[22] Castello A., Polla M., Buijsse B., Ruiz A., Casas A.M., Baena-Can J.M., Lope V., Antoli S., Ramos M., Mun M. (2014). Spanish Mediterranean diet and other dietary patterns and breast cancer risk: Case–control EpiGEICAM study. Br. J. Cancer.

[23] Haakensen V.D., Bjøro T., Lüders T., Riis M., Bukholm I.K., Kristensen V.N., Troester M.A., Homen M.M., Ursin G., Børresen-Dale A.L., Helland A. (2011). Serum oestradiol levels associated with specific gene expression patterns in normal breast tissue and in breast carcinomas. BMC Cancer.

[24] Yoshimoto N., Nishiyama T., Toyama T., Takahashi S., Shiraki N., Sugiura H., Endo Y., Iwasa M., Fujii Y., Yamashita H. (2011). Genetic and environmental predictors, endogenous hormones and growth factors, and risk of oestrogen receptor-positive breast cancer in Japanese women. Cancer Sci..

[25] Secreto G., Venturelli E., Meneghini E., Carcangiu M.L., Paolini B., Agresti R., Pellitteri C., Berrino F., Gion M., Cogliati P. (2012). Androgen receptors and serum testosterone levels identify different subsets of postmenopausal breast cancers. BMC Cancer.

[26] Hvidtfeldt U.A., Gunter M.J., Lange T., Chlebowski R.T., Lane D., Farhat G.N., Freiberg M.S., Keiding N., Lee J.S., Prentice R. (2012). Quantifying mediating effects of endogenous oestrogen and insulin in the relation between obesity, alcohol consumption, and breast cancer. Cancer Epidemiol. Biomarkers Prev..

[27] Kaaks R., Tikk K., Sookthai D., Schock H., Johnson T., Tjønneland A., Olsen A., Overvad K., Clavel-Chapelon F., Dossus L. (2014). Premenopausal serum sex hormone levels in relation to breast cancer risk, overall and by hormone receptor status—results from the EPIC cohort. Int. J. Cancer.

[28] Nyante S.J., Faupel-Badger J.M., Sherman M.E., Pfeiffer R.M., Gaudet M.M., Falk R.T., Andaya A.A., Lissowska J., Brinton L.A., Peplonska B. (2011). Genetic variation in PRL and PRLR, and relationships with serum prolactin levels and breast cancer risk: results from a population based case-control study in Poland. Breast Cancer Res..

[29] Flint M.S., Bovbjerg D.H. (2012). DNA damage as a result of psychological stress: implications for breast cancer. Breast Cancer Res..

[30] Zeitzer J.M., Nouriani B., Rissling M.B., Sledge G.W., Kaplan K.A., Aasly L., Palesh O., Jo B., Neri E., Dhabhar F.S., Spiegel D. (2016). Aberrant nocturnal cortisol and disease progression in women with breast cancer. Breast Cancer Res. Treat..

[31] Allott E.H, Hursting S.D. (2015). Obesity and cancer: mechanistic insights from transdisciplinary studies. Endocr. Relat. Cancer..

[32] Baek A.E., Nelson E.R. (2016). The Contribution of Cholesterol and its Metabolites to the Pathophysiology of Breast Cancer. Horm. Cancer..

[33] Agnoli C., Grioni S., Sieri S., Sacerdote C., Ricceri F., Tumino R., Frasca G., Pala V., Mattiello A., Chiodini P., Iacoviello L., De Curtis A., Panico S., Krogh V. (2015). Metabolic syndrome and breast cancer risk: a case-cohort study nested in a multicentre Italian cohort. PLoS ONE.

[34] Ni H., Liu H., Gao R. (2015). Serum lipids and breast cancer risk: a meta-analysis of prospective cohort studies. PLoS ONE.

[35] Touvier M., Fassier P., His M., Norat T., Chan D.S.M., Blacher J., Hercberg S., Galan P., Druesne-Pecollo N., Latino-Martel P. (2015). Cholesterol and breast cancer risk: a systematic review and meta-analysis of prospective studies. Brit. J. Nutr..

[36] Borgquist S., Butt T., Almgren P., Shiffman D., Stocks T., Orho-Melander M., Manjer J., Melander O. (2016). Apolipoproteins, lipids and risk of cancer. Int. J. Cancer.

[37] Agnoli C., Berrinob F., Abagnatoc C.A., Mutid P., Panicoe S., Crosignanif P., Krogha V. (2010). Metabolic syndrome and postmenopausal breast cancer in the ORDET cohort: a nested case-control study. Nutr. Metab. Cardiovasc. Dis..

[38] Kabat G.C., Kim M., Chlebowski R.T., Khandekar J., Ko M.G., McTiernan A., Neuhouser M.L., Parker D.R., Shikany J.M., Stefanick M.L. (2009). A longitudinal study of the metabolic syndrome and risk of postmenopausal breast cancer. Cancer Epidemiol. Biomarkers Prev..

[39] World Health Organization, International Agency for Research on Cancer International Classification of Diseases for Oncology ICD-O-3 online. http://codes.iarc.fr/topography/C50.

[40] Krusinska B., Hawrysz I., Wadolowska L., Slowinska M.A., Biernacki M., Czerwinska A., Golota J.J. (2018). Associations of Mediterranean diet and a posteriori derived dietary patterns with breast and lung cancer risk: a case-control study. Nutrients.

[41] Lidia Wadolowska. http://codes.iarc.fr/topography/C50.

[42] Fung T.T., McCullough M.L., Newby P.K., Manson J.E., Meigs J.B., Rifai N., Willett W.C., Hu F.B. (2005). Diet-quality scores and plasma concentrations of markers of inflammation and endothelial dysfunction. Am. J. Clin. Nutr..

[43] (2002). National Cholesterol Education Program (NCEP) Expert panel on Detection, Evaluation, and Treatment of High Blood Cholesterol in Adults (Adult Treatment Panel III). Circulation.

[44] (2016). 2016 European Guidelines on cardiovascular disease prevention in clinical practice: The Sixth Joint Task Force of the European Society of Cardiology and Other Societies on Cardiovascular Disease Prevention in Clinical Practice (constituted by representatives of 10 societies and by invited experts) Developed with the special contribution of the European Association for Cardiovascular Prevention & Rehabilitation (EACPR). Atherosclerosis.

[45] Armitage P., Berry G., Matthews J.N.S. (2001). Statistical Methods in Medical Research.

[46] Previdelli Á.N., de Andrade S.C., Fisberg R.M., Marchioni D.M. (2016). Using two different approaches to assess dietary patterns: Hypothesis-driven and data-driven analysis. Nutrients.

[47] Falk R.T., Gentzschein E., Stanczyk F.Z., Garcia-Closas M., Figueroa J.D., Ioffe O.B., Lissowska J., Brinton L.A., Sherman M.E. (2012). Sex steroid hormone levels in breast adipose tissue and serum in postmenopausal women. Breast. Cancer Res. Treat..

[48] Widschwendter M., Rosenthal A.N., Philpott S., Rizzuto I., Fraser L., Hayward J., Intermaggio M.P., Edlund Ch.K., Ramus S.J., Gayther S.A. (2013). The sex hormone system in carriers of BRCA1/2 mutations: A case-control study. Lancet Oncol..

[49] Tworoger S.S., Hankinson S.E. (2008). Prolactin and breast cancer etiology: an epidemiologic perspective. J. Mammary Gland. Biol. Neoplasia.

[50] Wang M., Wu X., Chai F., Zhang Y., Jiang J. (2016). Plasma prolactin and breast cancer risk: A meta- analysis. Sci. Rep..

[51] Sprague B.L., Trentham-Dietz A., Gangnon R.E., Buist D.S.M., Burnside E.S., Bowles E.J.A., Stanczyk F.Z., Sisney G.S. (2011). Circulating sex hormones and mammographic breast density among postmenopausal women. Horm. Cancer.

[52] McHale K., Tomaszewski J.E., Puthiyaveettil R., LiVolsi V.A., Clevenger Ch.V. (2008). Altered expression of prolactin receptor associated signalling proteins in human breast carcinoma. Mod. Pathol..

[53] Esposito K., Chiodini P., Capuano A., Bellastella G., Maiorino M., Rafaniello C., Giugliano D. (2013). Metabolic syndrome and postmenopausal breast cancer: systematic review and meta-analysis. Menopause.

[54] Alokail M.S., Al-Daghri N., Abdulkareem A., Draz H.M., Yakout S.M., Alnaami A.M., Sabico S., Alenad A.M., Chrousos G.P. (2013). Metabolic syndrome biomarkers and early breast cancer in Saudi women: Evidence for the presence of a systemic stress response and/or a pre-existing metabolic syndrome-related neoplasia risk?. BMC Cancer.

[55] Melvin J.C., Garmo H., Holmberg L., Hammar N., Walldius G., Jungner I., Lambe M., Van Hemelrijck M. (2017). Glucose and lipoprotein biomarkers and breast cancer severity using data from the Swedish AMORIS cohort. BMC Cancer.

[56] Nelson E.R., Chang Ch., McDonnell D.P. (2014). Cholesterol and breast cancer pathophysiology. Trends Endocrinol. MeTable.

[57] Kabat G.C., Kim M., Caan B.J., Chlebowski R.T., Gunter M.J., Ho G.Y.F., Rodriguez B.L., Shikany J.M., Strickler H.D., Vitolins M.Z. (2009). Repeated measures of serum glucose and insulin in relation to postmenopausal breast cancer. Int. J. Cancer.

[58] Sieri S., Agnoli C., Pala V., Mattiello A., Panico S., Masala G., Assedi M., Tumino R., Frasca G., Sacerdote C., Vineis P., Krogh V. (2015). Dietary habits and cancer: the experience of EPIC-Italy. Epidemiol. Prev..

[59] Norat T., Scoccianti C., Boutron-Ruault M.C., Anderson A., Berrino F., Cecchini M., Espina C., Key T., Leitzmann M., Powers H. (2015). European Code against Cancer 4th Edition: Diet and cancer. Cancer Epidemiol..

[60] Mourouti N., Papavagelis C., Plytzanopoulou P., Kontogianni M., Vassilakou T., Malamos N., Linos A., Panagiotakos D. (2015). Dietary patterns and breast cancer: a case-control study in women. Eur. J. Nutr..

[61] Van den Brandt P.A., Schulpen M. (2017). Mediterranean diet adherence and risk of postmenopausal breast cancer: Results of a cohort study and meta-analysis. Int. J. Cancer.

[62] Turati F., Carioli G., Bravi F., Ferraroni M., Serraino D., Montella M., Giacosa A., Toffolutti F., Negri E., Levi F., La Vecchia C. (2018). Mediterranean diet and breast cancer risk. Nutrients.

[63] Penniecook-Sawyers J.A., Jaceldo-Siegl K., Fan J., Beeson L., Knutsen S., Herring P., Fraser G.E. (2016). Vegetarian dietary patterns and the risk of breast cancer in a low-risk population. Br. J. Nutr..

[64] Song J.W., Chung K.C. (2010). Observational studies: cohort and case-control studies. Plast. Reconstr. Surg..

