# Supplementary material for: Associations of Dietary Patterns and Metabolic-Hormone Profiles with Breast Cancer Risk: A Case-Control Study
Source: Nutrients. 2018 Dec 19;10(12):2013. doi: 10.3390/nu10122013 (PMC6316263; doi:10.3390/nu10122013)
Supplement: Supplementary file 1 [file nutrients-10-02013-s001.pdf]

Table S1. The mean (95% CI) of the frequency of food consumption by dietary patterns for cancer-control sample (times/day).

Food groups[#]	Total	**Dietary Patterns** 'Non-Healthy' tertiles			'Prudent' tertiles			'Margarine and Sweetened Dairy' tertiles			'Polish-aMED' levels		
		bottom	middle	upper	bottom	middle	upper	bottom	middle	upper	low	average	high
Sample size (n)	420	139	141	140	139	140	141	140	139	141	51	224	145
Sugar, honey and sweets	1.9 (1.7; 2.0)	1.2 (1.0; 1.3)	1.7 (1.5; 1.8)	2.8 (2.6; 3.0)	1.7 (1.5; 1.9)	1.9 (1.7; 2.1)	2.1 (1.8; 2.3)	2.0 (1.7; 2.2)	1.7 (1.5; 1.9)	2.0 (1.8; 2.2)	2.1 (1.7; 2.4)	2.0 (1.8; 2.2)	1.6 (1.5; 1.8)
Red and processed meats	1.4 (1.3; 1.4)	0.8 (0.7; 0.9)	1.4 (1.3; 1.5)	1.9 (1.8; 2.0)	1.3 (1.2; 1.4)	1.3 (1.2; 1.5)	1.5 (1.3; 1.6)	1.4 (1.3; 1.5)	1.3 (1.1; 1.4)	1.4 (1.3; 1.5)	1.7 (1.5; 1.9)	1.5 (1.4; 1.6)	1.0 (0.9; 1.2)
Animal fats	1.1 (1.1; 1.2)	0.7 (0.6; 0.8)	1.1 (1.0; 1.2)	1.6 (1.5; 1.8)	1.0 (0.9; 1.1)	1.2 (1.1; 1.4)	1.2 (1.1; 1.4)	1.9 (1.7; 2.0)	1.0 (0.9; 1.2)	0.5 (0.5; 0.6)	1.5 (1.2; 1.7)	1.3 (1.2; 1.4)	0.8 (0.7; 0.9)
Milk, fermented milk drinks and cheese curd	1.1 (1.0; 1.1)	1.2 (1.0; 1.3)	1.0 (0.9; 1.1)	1.1 (1.0; 1.2)	0.7 (0.6; 0.8)	1.1 (1.0; 1.2)	1.4 (1.3; 1.6)	0.9 (0.8; 1.0)	1.2 (1.1; 1.3)	1.1 (1.0; 1.2)	0.8 (0.6; 0.9)	1.0 (0.9; 1.1)	1.3 (1.1; 1.4)
Refined cereals	0.9 (0.9; 1.0)	0.4 (0.3; 0.4)	0.9 (0.8; 1.0)	1.5 (1.4; 1.6)	1.2 (1.1; 1.3)	0.8 (0.7; 0.9)	0.8 (0.7; 0.9)	0.9 (0.8; 1.1)	0.8 (0.7; 0.9)	1.1 (1.0; 1.2)	1.5 (1.3; 1.7)	1.0 (0.9; 1.1)	0.6 (0.5; 0.7)
Vegetables	1.2 (1.2; 1.3)	1.2 (1.1; 1.3)	1.2 (1.2; 1.3)	1.2 (1.1; 1.3)	1.0 (0.9; 1.1)	1.2 (1.1; 1.3)	1.5 (1.4; 1.6)	1.3 (1.3; 1.4)	1.2 (1.1; 1.3)	1.1 (1.0; 1.2)	0.9 (0.8; 1.0)	1.2 (1.1; 1.2)	1.4 (1.3; 1.5)
Fruit	1.0 (0.9; 1.0)	1.0 (0.9; 1.1)	1.0 (0.9; 1.0)	0.9 (0.8; 1.0)	0.7 (0.6; 0.8)	0.9 (0.9; 1.0)	1.3 (1.2; 1.3)	1.0 (0.9; 1.1)	1.0 (0.9; 1.1)	1.0 (0.9; 1.0)	0.7 (0.5; 0.8)	0.9 (0.8; 1.0)	1.2 (1.1; 1.2)
Wholemeal cereals	0.9 (0.8; 1.0)	1.3 (1.2; 1.4)	0.9 (0.7; 1.0)	0.5 (0.4; 0.6)	0.5 (0.4; 0.6)	0.9 (0.8; 1.0)	1.3 (1.1; 1.4)	0.9 (0.8; 1.0)	1.0 (0.8; 1.1)	0.8 (0.7; 0.9)	0.3 (0.1; 0.4)	0.8 (0.7; 0.9)	1.3 (1.1; 1.4)
Potatoes	0.6 (0.6; 0.7)	0.4 (0.3; 0.4)	0.7 (0.6; 0.7)	0.8 (0.8; 0.8)	0.7 (0.6; 0.7)	0.6 (0.5; 0.6)	0.6 (0.6; 0.7)	0.7 (0.6; 0.7)	0.6 (0.5; 0.6)	0.6 (0.6; 0.7)	0.6 (0.6; 0.7)	0.7 (0.6; 0.7)	0.5 (0.5; 0.6)
Vegetable oils	0.6 (0.6; 0.6)	0.5 (0.5; 0.6)	0.6 (0.6; 0.7)	0.7 (0.7; 0.8)	0.5 (0.4; 0.5)	0.7 (0.6; 0.7)	0.7 (0.7; 0.8)	0.6 (0.6; 0.7)	0.6 (0.5; 0.6)	0.6 (0.6; 0.7)	0.5 (0.4;0.6)	0.6 (0.6; 0.7)	0.6 (0.6; 0.7)
Other fats	0.5 (0.4; 0.5)	0.3 (0.2; 0.4)	0.5 (0.4; 0.6)	0.6 (0.5; 0.7)	0.6 (0.5; 0.7)	0.3 (0.2; 0.4)	0.5 (0.4; 0.6)	0.1 (0.1; 0.1)	0.2 (0.1; 0.2)	1.1 (1.0; 1.2)	0.5 (0.3; 0.7)	0.5 (0.4; 0.6)	0.4 (0.3; 0.5)
Cheese	0.4 (0.3; 0.4)	0.3 (0.3; 0.4)	0.4 (0.3; 0.4)	0.4 (0.4; 0.5)	0.3 (0.2; 0.3)	0.3 (0.3; 0.4)	0.5 (0.4; 0.5)	0.4 (0.3; 0.4)	0.3 (0.3; 0.4)	0.4 (0.4; 0.5)	0.4 (0.3; 0.4)	0.4 (0.3; 0.4)	0.4 (0.3; 0.4)
White meat	0.4 (0.4; 0.5)	0.4 (0.3; 0.4)	0.5 (0.4; 0.5)	0.5 (0.5; 0.5)	0.4 (0.4; 0.5)	0.4 (0.4; 0.5)	0.5 (0.4; 0.5)	0.4 (0.3; 0.4)	0.5 (0.4; 0.5)	0.5 (0.5; 0.6)	0.4 (0.4; 0.5)	0.5 (0.4; 0.5)	0.4 (0.4; 0.5)
Juices	0.5 (0.4; 0.5)	0.3 (0.3; 0.4)	0.5 (0.4; 0.6)	0.6 (0.5; 0.7)	0.2 (0.2; 0.3)	0.4 (0.3; 0.5)	0.8 (0.7; 0.9)	0.5 (0.4; 0.6)	0.5 (0.4; 0.6)	0.4 (0.4; 0.5)	0.4 (0.3; 0.5)	0.5 (0.4; 0.5)	0.5 (0.4; 0.6)
Eggs	0.4 (0.3; 0.4)	0.3 (0.3; 0.3)	0.3 (0.3; 0.4)	0.4 (0.4; 0.5)	0.2 (0.2; 0.3)	0.3 (0.3; 0.4)	0.5 (0.5; 0.6)	0.4 (0.4; 0.5)	0.3 (0.3; 0.4)	0.3 (0.3; 0.4)	0.3 (0.3; 0.4)	0.3 (0.3; 0.4)	0.4 (0.3; 0.4)
Sweetened milk beverages and flavored cheese curds	0.3 (0.3; 0.4)	0.2 (0.1; 0.2)	0.3 (0.2; 0.4)	0.5 (0.4; 0.6)	0.2 (0.1; 0.2)	0.3 (0.2; 0.4)	0.5 (0.4; 0.6)	0.2 (0.1; 0.2)	0.3 (0.2; 0.4)	0.5 (0.4; 0.6)	0.3 (0.2; 0.4)	0.3 (0.3; 0.4)	0.3 (0.2; 0.3)
Fish	0.3 (0.2; 0.3)	0.3 (0.2; 0.4)	0.3 (0.2; 0.3)	0.3 (0.2; 0.3)	0.1 (0.1; 0.2)	0.2 (0.2; 0.3)	0.5 (0.4; 0.5)	0.2 (0.2; 0.3)	0.3 (0.2; 0.3)	0.3 (0.2; 0.3)	0.1 (0.1; 0.1)	0.2 (0.2; 0.3)	0.4 (0.3; 0.4)
Nuts and seeds	0.3 (0.3; 0.4)	0.5 (0.4; 0.6)	0.3 (0.2; 0.3)	0.2 (0.1; 0.2)	0.1 (0.1; 0.2)	0.3 (0.2; 0.4)	0.6 (0.5; 0.7)	0.4 (0.3; 0.5)	0.4 (0.3; 0.5)	0.3 (0.2; 0.3)	0.0 (0.0; 0.0)	0.2 (0.2; 0.3)	0.6 (0.5; 0.7)

Table S1. *Cont.*

Food groups[#]	Total	Dietary Patterns											
		**'Non-Healthy' tertiles**			**'Prudent' tertiles**			**'Margarine and Sweetened Dairy' tertiles**			**'Polish-aMED' levels**		
		bottom	middle	upper	bottom	middle	upper	bottom	middle	upper	low	average	high
Sample size (n)	420	139	141	140	139	140	141	140	139	141	51	224	145
Legumes	0.2 (0.2; 0.2)	0.2 (0.2; 0.3)	0.2 (0.2; 0.2)	0.2 (0.2; 0.3)	0.1 (0.1; 0.1)	0.2 (0.1; 0.2)	0.4 (0.3; 0.4)	0.2 (0.1; 0.2)	0.2 (0.2; 0.3)	0.3 (0.2; 0.3)	0.1 (0.0; 0.1)	0.2 (0.1; 0.2)	0.3 (0.3; 0.4)
Breakfast cereals	0.2 (0.1; 0.2)	0.1 (0.1; 0.2)	0.2 (0.1; 0.2)	0.2 (0.1; 0.2)	0.0 (0.0; 0.1)	0.2 (0.1; 0.2)	0.3 (0.2; 0.3)	0.1 (0.0; 0.1)	0.2 (0.1; 0.2)	0.3 (0.2; 0.3)	0.1 (0.0; 0.1)	0.2 (0.1; 0.2)	0.2 (0.1; 0.3)
Sweetened beverages and energy drinks	0.1 (0.0; 0.1)	0.0 (0.0; 0.0)	0.0 (0.0; 0.0)	0.1 (0.1; 0.2)	0.0 (0.0; 0.1)	0.0 (0.0; 0.1)	0.1 (0.1; 0.1)	0.0 (0.0; 0.1)	0.0 (0.0; 0.1)	0.1 (0.1; 0.1)	0.1 (0.0; 0.1)	0.1 (0.0; 0.1)	0.1 (0.0; 0.1)

'Polish-aMED' – 'Polish-adapted Mediterranean Diet' (range of points: 0-8), levels (in points): 'low' (0-2), 'average' (3-5), 'high' (6-8); [#]the frequency consumption was expressed as a times/day after assigning the values for categories of frequency consumption as follows: 'never or almost never'=0; 'once a month or less'=0.025; 'several times a month'=0.1; 'several times a week'=0.571; 'daily'=1; 'several times a day'=2; 95%CI – 95% confidence interval.

Table S2. Description of food groups for the 'Polish-adapted Mediterranean Diet' score (0-8 points) calculation – data for the Initial control sample (n=242).

Food groups/food items	Frequency of consumption (times/day)*		Criteria for 1 point
	**Mean (95% CI)**	**Median**	
**VEGETABLES**: all kinds of vegetables: cruciferous vegetables (cabbages, brussel sprouts, cauliflower, broccoli, kale, etc.), yellow-orange vegetables (carrots, peppers, etc.), leafy green vegetables (spinach, chicory, lettuce, rocket, leek, celery, parsley, etc.), tomatoes, gourds and squashes (fresh cucumber, marrow, courgettes, pumpkins, aubergines, etc.), root vegetables and other (parsnip, beetroots, onion, garlic, celeriac, radishes, turnip, salads and mixed vegetables, etc.)	1.300 (1.230; 1.371)	1.000	Greater than median intake (times/day)*
**FRUIT**: all kinds of fruit: stone fruit (apricots, cherries, nectarines, peaches, plums, grapes, etc.), kiwi and citrus fruit (kiwi, oranges, mandarins, grapefruit, lemons, pomelos, etc.), tropical fruits (pineapples, watermelon, melons, fresh dates and figs, etc.), berries (strawberries, raspberries, blackberries, blueberries, redcurrants, blackcurrants, etc.), bananas, apples, pears, avocado	0.989 (0.925; 1.052)	1.000	Greater than median intake (times/day)*
**WHOLEMEAL CEREALS**: wholemeal wheat or rye bread, seeded loafs, pumpernickel, wholemeal groats, wholemeal rice, wholemeal pasta	0.890 (0.799; 0.981)	0.671	Greater than median intake (times/day)*
**FISH**: lean fish (ollock, cod, perch, hake, carp to 1 kg, tuna, panga, trout, etc.) oily fish (salmon, sardines, herring, mackerel, eel, large carp, etc.)	0.293 (0.253; 0.334)	0.200	Greater than median intake (times/day)*
**LEGUMES**: fresh and tinned legumes (corn, green peas, green beans, etc.), dry and processed pulses beans (fava, broad, French, green), soya, peas, chickpea and processed pulses (baked beans, hummus, other bread spreads)	0.223 (0.187; 0.259)	0.125	Greater than median intake (times/day)*
**NUTS** and **SEEDS**: peanuts, hazelnuts, walnuts, almonds, pistachios, cashews, coconuts, chestnuts, etc., pumpkin seeds, sesame seeds, sunflower seeds, wheat germs, etc.	0.375 (0.312; 0.438)	0.125	Greater than median intake (times/day)*
**RATIO of VEGETABLES OILS** (rapeseed oil, sunflower oil, linseed oil, olives) **to ANIMAL FAT** (butter, cream, lard) instead of RATIO of MONOUNSATURATED to SATURATED FAT	1.462 (1.125; 1.799)	0.519	Greater than median intake (times/day)*
**RED** and **PROCESED MEAT**: red meat (pork, beef, veal, etc.), sausages, bacon, reconstituted meat (sausages, meat loaf, hot-dogs, smoked sausages, bacon, etc.), high quality cured meats (ham, poultry and pork-beef good quality cold meats, etc.), offal products (liver, blood sausage, sweetbread, liver pate, etc.), game (wild boar, venison, quail, mallard, hare, etc.)	1.252 (1.151; 1.352)	1.267	Lower than median intake (times/day)*

*food frequency consumption was expressed as a times/day after assigning the values for categories of frequencies as follows: 'never or almost never'=0; 'once a month or less'=0.025; 'several times a month'=0.1; 'several times a week'=0.571; 'daily'=1; 'several times a day'=2; 95% CI – 95% confidence interval.

Table S3. Confounders in the case-control study regarding association of dietary patterns and metabolic-hormone profiles with breast cancer risk.

Confounders	Categories
**Age** (years)	40.0-49.9; 50.0-59.9; 60.0-69.9; 70.0-79.9
**Age at menarche** (years)	<12.0; 12-14.9; ≥15.0
**Menopausal status**	premenopausal; postmenopausal
**Oral contraceptive use** (ever)	no; yes
**Hormone-replacement therapy use**	no; yes
**Number of children**	0; 1-2; ≥3
**BMI** (kg/m^2)<br>calculated using measured weight and height	≤24.9; 25.0-29.9; ≥30.0
**Socioeconomic status (SES)**<br>after combining data based on SES' factors:	low; average; high
place of residence	village
	town <20,000 inhabitants
	town 20,000-100,000 inhabitants
	city >100,000 inhabitants
educational level	primary
	secondary
	higher
economic situation<br>(self-declared)	below average
	average
	above average
situation of household (self-declared)	we live poorly – I don't have enough resources even for basic needs (food/clothing/housing fees)<br>we live very thriftily – I have enough resources only for basic needs (food/clothing/housing fees)<br>we live thriftily – so I have enough resources for everything<br>we live well – I have enough resources for everything, but I don't put off savings<br>we live very well – I have enough resources for everything and I put off savings

Table S3. *Cont.*

**Overall physical activity** after combining data based on physical activity at work and physical activity in leisure time	low; moderate; high	
physical activity at work	low	more than 70% of working time spent sedentary or retired
	moderate	approx. 50% of working time spent sedentary and 50% of working time spent in an active manner
	high	approx. 70% of working time spent in an active manner or physical work related to great exertion
physical activity in leisure time	low	sedentary for most of the time, watching TV, reading books, walking 1-2 hours per week
	moderate	walking, bike riding, gymnastics, gardening, light physical activity performed 2-3 hours per week
	high	bike riding, jogging, gardening, sport activities involving physical exertion performed more than 3 hours weekly
**Abuse of alcohol**	no	
	yes	intake at least 1 bottle (0.5 liters) of beer or 2 glasses of wine (300ml) or 2 drinks (300ml) or 2 glasses of vodka (60ml) per day
**Smoking status**	non-smoker	
	smoker	current-smoker or/ and former-smoker
**Vitamin/mineral supplements use** (within last 12 months)	no; yes	
**Family history of breast cancer in first- or second-degree relative**	no; I don't know; yes	
**Molecular of breast cancer subtypes**	triple negative; ER-, PR-, HER2+ subtype; luminal A; luminal B	

Table S4. 'High-Hormone' profile, serum hormone concentration and metabolic syndrome components by 'Metabolic-Syndrome' profile (%).

Variable	**'Metabolic-Syndrome' Profile** (tertiles)			*p*-Value
	**bottom**	**middle**	**upper**	
Sample Size	43	43	43	
Hormones				
estradiol (pg/mL)*	15.8 (57.0)	11.0 (15.6)	14.8 (36.7)	ns
progesterone (ng/mL)*	0.19 (0.64)	0.18 (0.33)	0.11 (0.14)	ns
prolactin (ng/mL)*	10.7 (4.0)	13.9 (17.0)	19.0 (30.9)	ns
testosterone (ng/mL)*	0.16 (0.09)[ab]	0.21 (0.11)[a]	0.22 (0.15)[b]	ns
cortisol (μg/dL)*	15.6 (4.9)	15.6 (7.8)	15.3 (7.2)	ns
insulin (μU/mL)*	6.4 (2.4)[ab]	9.9 (5.9)[ac]	14.2 (10.0)[bc]	<0.0001
Metabolic syndrome biomarkers				
triglycerides (mg/dL)*	81.0 (23.8)[a]	90.1 (24.9)[b]	141.1 (64.5)[ab]	<0.0001
<150	100.0[a]	100.0[b]	64.3[ab]	<0.0001
≥150	0.0[c]	0.0[d]	35.7[cd]	
HDL-cholesterol (mg/dL)*	81.2 (16.6)[ab]	68.8 (10.5)[ac]	52.3 (10.4)[bc]	<0.0001
≥50	100.0[a]	95.3[b]	59.5[ab]	<0.0001
<50	0.0[c]	4.7[d]	40.5[cd]	
glucose (mg/dL)*	91.8 (9.4)[ab]	96.4 (10.3)[a]	100.3 (11.8)[b]	0.0018
<100	79.1[a]	72.1[b]	47.6[ab]	0.0056
≥100	20.9[c]	27.9[d]	52.4[cd]	
hypertension (self-reported)	0.0[ab]	20.9[ac]	61.9[bc]	<0.0001
waist circumference (cm)*	78.2 (6.9)[ab]	88.9 (8.4)[ac]	99.1 (10.7)[bc]	<0.0001
<88	93.0[ab]	41.9[ac]	16.7[bc]	<0.0001
≥88	7.0[de]	58.1[df]	83.3[ef]	
Metabolic syndrome Score (MetS)*	0.3 (0.5)[ab]	1.1 (0.8)[ac]	2.7 (1.0)[bc]	<0.0001
0	72.1[ab]	18.6[ac]	0.0[bc]	
1-2	27.9[de]	76.7[df]	47.6[ef]	<0.0001
3-5	0.0[g]	4.7[h]	52.4[gh]	
without metabolic syndrome (0-2)	100.0[a]	95.3[b]	47.6[ab]	<0.0001
with metabolic syndrome (3-5)	0.0[c]	4.7[d]	52.4[cd]	
total cholesterol (mg/dL)*	229.1 (36.9)[ab]	208.5 (36.7)[a]	203.3 (47.6)[b]	0.0097
LDL-cholesterol (mg/dL)*	134.1 (32.6)	121.7 (31.8)	123.6 (44.6)	ns
log TG/HDL*	0.0 (0.2)[ab]	0.1 (0.1)[ac]	0.4 (0.2)[bc]	<0.0001
<0.50	100.0[a]	100.0[b]	69.0[ab]	<0.0001
≥0.50	0.0[c]	0.0[d]	31.0[cd]	
LDL/HDL*	1.7 (0.5)[a]	1.8 (0.5)[b]	2.5 (1.2)[ab]	0.0001
<3.50	100.0[a]	100.0[b]	83.3[ab]	0.0005
≥3.50	0.0[c]	0.0[d]	16.7[cd]	
non-HDL (mg/dL)*	148.0 (32.8)	139.7 (32.7)	151.0 (48.3)	ns
<145	46.5	53.5	45.2	ns
≥145	53.5	46.5	54.8	

HDL – high-density lipoprotein; LDL – low-density lipoprotein; TG – triglycerides; % – sample percentage; *mean and standard deviation (SD); *p*-value – level of significance verified with by chi^2 test (categorical variables) or Kruskal-Wallis' test (continuous variables) or Student's t-test (for log-transformed serum

biomarkers concentration); a-a, ..., h-h – statistically significant differences between pairs of the 'Metabolic-Syndrome' profile tertiles, $p < 0.05$.

Table S5. 'Metabolic-Syndrome' profile, metabolic syndrome components and serum hormone concentration by 'High-Hormone' profile (%).

**Variable**	**'High-Hormone' Profile** (tertiles)			***p*-Value**
	**bottom**	**middle**	**upper**	
Sample Size	42	44	43	
'Metabolic-Syndrome' Profile				
*score**	104.4 (44.6)	113.3 (46.2)	116.3 (34.0)	ns
tertiles				
bottom	42.9[a]	40.9[b]	16.7[ab]	
middle	26.2	36.4	38.1	0.0443
upper	31.0	22.7[c]	45.2[c]	
Hormones				
estradiol (pg/mL)*	6.8 (4.3)[a]	7.7 (8.8)	27.5 (67.5)[a]	0.0006
progesterone (ng/mL)*	0.07 (0.02)[a]	0.08 (0.03)[b]	0.34 (0.71)[ab]	<0.0001
prolactin (ng/mL)*	10.5 (3.5)[a]	9.6 (3.5)[b]	23.7 (34.0)[ab]	0.0005
testosterone (ng/mL)*	0.10 (0.05)[ab]	0.18 (0.08)[ac]	0.31 (0.12)[bc]	<0.0001
cortisol (µg/dL)*	11.7 (3.8)[ab]	14.2 (4.8)[ac]	20.6 (7.6)[bc]	<0.0001
insulin (µU/mL)*	13.0 (10.9)[ab]	8.2 (4.1)[a]	9.4 (4.6)[b]	0.0305
Metabolic syndrome biomarkers				
triglycerides (mg/dL)*	98.3 (48.4)	108.2 (58.0)	104.6 (40.2)	ns
<150	90.5	86.4	88.1	ns
≥150	9.5	13.6	11.9	
HDL-cholesterol (mg/dL)*	76.1 (19.5)[ab]	65.4 (14.9)[a]	61.3 (14.3)[b]	0.0007
≥50	90.5	86.4	78.6	ns
<50	9.5	13.6	21.4	
glucose (mg/dL)*	99.5 (10.8)	93.7 (10.8)	95.3 (11.0)	0.0410
<100	52.4[a]	77.3[a]	69.0	0.0459
≥100	47.6[b]	22.7[b]	31.0	
hypertension (self-reported)	28.6	18.2	35.7	ns
waist circumference (cm)*	87.4 (11.7)	88.2 (12.0)	90.4 (13.0)	ns
<88	52.4	56.8	42.9	ns
≥88	47.6	43.2	57.1	
Metabolic syndrome Score (MetS)*	1.4 (1.4)	1.1 (1.3)	1.6 (1.1)	ns
0	33.3	40.9[a]	16.7[a]	
1-2	42.9	45.5	64.3	ns
3-5	23.8	13.6	19.0	
without metabolic syndrome (0-2)	76.2	86.4	81.0	ns
with metabolic syndrome (3-5)	23.8	13.6	19.0	
total cholesterol (mg/dL)*	218.9 (43.8)	218.3 (30.6)	203.7 (48.8)	ns
LDL-cholesterol (mg/dL)*	125.6 (38.1)	132.0 (30.4)	121.5 (41.5)	ns
log TG/HDL*	0.1 (0.3)	0.2 (0.3)	0.2 (0.2)	ns
<0.50	92.9	88.6	88.1	ns
≥0.50	7.1	11.4	11.9	

Table S5. *Cont.*

Variable	'High-Hormone' Profile (tertiles)			p-Value
	bottom	middle	upper	
Sample Size	42	44	43	
LDL/HDL*	1.7 (0.6)[ab]	2.2 (0.9)[a]	2.1 (0.9)[b]	0.0193
<3.50	100.0[a]	90.9[a]	92.9	ns
≥3.50	0.0[b]	9.1[b]	7.1	
non-HDL (mg/dL)*	142.9 (35.9)	152.9 (33.6)	142.4 (45.3)	ns
<145	52.4	38.6	54.8	ns
≥145	47.6	61.4	45.2	

HDL – high-density lipoprotein; LDL – low-density lipoprotein; TG – triglycerides; % – sample percentage; *mean and standard deviation (SD); *p*-value – level of significance verified with chi^2 test (categorical variables) or Kruskal-Wallis' test (continuous variables) or Student's t-test (for log-transformed serum biomarkers concentration); a-a, ..., c-c – statistically significant differences between pairs of the 'High-Hormone' profile tertiles, $p < 0.05$.
